# Evolving dimensions of women’s empowerment in India

**DOI:** 10.1371/journal.pone.0327494

**Published:** 2025-07-11

**Authors:** Bharti Singh, Shri K. Singh

**Affiliations:** Department of Survey Research and Data Analytics, International Institute for Population Sciences, Mumbai, India; University of California Los Angeles, UNITED STATES OF AMERICA

## Abstract

Empowerment is a dynamic, multifaceted concept shaped by intersecting socio-economic, cultural, and demographic contexts. The conceptualization of women’s empowerment has evolved from ensuring basic rights, such as education and employment, to addressing complexities that secure women’s rightful place in society, reflecting cultural and historical struggles for equality and inclusion. This study explores the evolving dimensions of women’s empowerment in India from 2006 to 2021, using data from three recent rounds of the National Family Health Survey. We employed Confirmatory Factor Analysis to investigate changes in the different dimensions of women’s empowerment and develop a robust empowerment index. To understand the effect of socio-economic factors on women’s empowerment, we conducted a Multinomial Logistic Regression analysis. Furthermore, Age-Period-Cohort (APC) analysis was performed to capture temporal and generational evolution of women’s empowerment and its dimensions. Our analysis revealed shifts in key dimensions of women’s empowerment over time. In 2005–06, “Freedom of Mobility” emerged as the dominant dimension, whereas “Financial Independence” became prominent in 2015–16, and “Attitude towards Violence” took precedence by 2019–21. During 2005–06, older women (40–49 years) exhibited a higher level of empowerment compared to younger women (15–30 years). However, by 2015–16, the concentration of more empowered women shifted to younger women (25–35 years). APC analysis indicated that age plays a significant role in women’s empowerment, with women approximately 30 years of age exhibiting the highest levels of empowerment. Additionally, older cohorts (born before 1970) showed no significant effects on empowerment. In contrast, cohorts born after 1970 demonstrated a significant impact.

## Introduction

Women’s empowerment is a complex and dynamic concept that has been the centre of research for examining the position of women in society. Despite its importance in assessing and addressing gender inequality, serving as a pathway to achieving SDG goals and fostering societal progress, women continue to struggle for their rightful place in society [1–4]. Women’s empowerment goes beyond mere economic independence or participation in decision-making. It encompasses a broad spectrum of rights, opportunities, and social changes woven with a socio-cultural and political fabric that enables women to live with dignity, equality, and freedom [3].

The definition of women’s empowerment has evolved significantly, reflecting the shift in global development priorities. Initially, empowerment was framed through a rights-based lens. Early definitions emphasized legal entitlements and reproductive autonomy. The International Conference on Population and Development (ICPD), Cairo, 1994, marked a key shift, with the United Nations Population Fund (UNFPA) identifying reproductive rights and health as central to women’s empowerment [5]. This view placed bodily autonomy and informed choice at the core of empowerment.

In the late 1990s, Amartya Sen’s Capabilities Approach (1999) expanded the discourse by redefining development as the expansion of individuals’ substantive freedoms to pursue the life they value. He argued that empowerment requires not only economic resources but also the real ability to pursue valued goals such as health, education, and political participation [6]. Expanding on this, Naila Kabeer (1999) conceptualized empowerment as a process of gaining the ability to make strategic life choices in contexts where it was previously denied. Her framework includes three dimensions: resources (material, social, and human), agency (capacity to define and act on one’s goals), and achievements (outcomes of those choices). Kabeer’s model is often seen as distinct, but she acknowledges that Sen’s capabilities can be understood as a combination of agency and resources [7], highlighting their conceptual alignment. Further, Martha Nussbaum’s (2000) version of the capabilities approach proposed a list of ten central capabilities essential for gaining empowerment, including bodily health, integrity, affiliation, and control over one’s environment. She emphasized that empowerment requires increasing capabilities while addressing systemic power inequalities [8]. Subsequent frameworks, such as Malhotra et al. (2002), emphasized the multidimensionality of empowerment, including economic, social, legal, and psychological domains [9]. Recent extensions include intersectionality [10], collective agency [11], and digital empowerment [12].

In addition to these theoretical frameworks, several indices have been constructed and modified to measure women’s empowerment and its dynamic nature over time. The UNDP introduced the Gender-related Development Index (GDI) and Gender Empowerment Measure (GEM), with GEM being the first index to measure gender inequality based on women’s relative income [13]. By 2010, these were combined into the Gender Inequality Index [14]. In 2017, a Survey-Based Women’s Empowerment index (SWPER) was developed and validated using the Demographic and Health Surveys, offering individual-level indicators of women’s empowerment, which also enables cross-country comparisons [15,16]. More recently, scholars have focused on the empirical challenges of measuring empowerment in context-specific ways. Seymour and Peterman (2018) highlighted how the meaning of intra-household decision-making varies across cultural settings, cautioning against universal assumptions in survey indicators [17]. Laszlo et al. (2020) critically examined the limitations of capturing economic empowerment in intra-household settings, especially regarding control over income and assets [18]. Peterman et al. (2021) explored how different survey designs and indicator choices shape the measurement of decision-making in cash and food transfer programs across Ecuador, Uganda, and Yemen, underscoring the importance of methodological rigor [19]. Sinharoy et al. (2023) developed the ARISE Scales, a context-specific tool for measuring women’s empowerment in urban sanitation across low- and middle-income countries, bringing attention to sectoral empowerment [20].

The evolution of women’s empowerment is also affected by socio-economic, demographic, ecological, and cultural factors varying across time and regions. It responds to structural changes such as economic development, education expansion, and policy interventions. The socio-economic status of women, particularly in terms of education and employment, plays a pivotal role in determining their autonomy and access to resources [21]. However, social stratification based on caste and wealth continues to produce unequal patterns of empowerment, with Scheduled Caste and Scheduled Tribe women often facing compounded disadvantages [22].

Demographic factors, including age, marital status, and number of children, significantly influence the degree of empowerment. Young married women, particularly those who enter cohabitation at an early age, frequently experience control by older family members, especially prevalent in patriarchal family structures [23]. Conversely, as women age and accumulate experience, their participation in household decision-making tends to increase. Cultural norms, including religion, kinship systems, and community traditions, significantly mediate empowerment outcomes [24–26]. However, the role of religion is affected by region, socio-economic class, and local customs. For instance, Muslim women in Kerala often show higher empowerment than Hindu women in Bihar, reflecting the role of state-level development and education [27]. The urban-rural divide further reflects disparities, with urban women being more likely to have better access to education, media, and health services.

Despite the progress in social status, women in India still face longstanding barriers including gender inequality, domestic violence, and mobility restrictions [26,28–30]. India’s complex social structure and patriarchal norms present unique challenges for defining and measuring women’s empowerment. Existing studies on women’s empowerment in India often adopt a static approach, analyzing empowerment at a single point in time or focusing on specific dimensions such as decision-making, freedom of mobility, or financial independence, without capturing the interconnectedness of these dimensions [21,24,31,32]. Although these studies provide valuable insights, they do not account for the temporal and multidimensional nature of empowerment in India. Some studies attempt to consolidate these dimensions into a composite measure, but they often treat all variables equally, which significantly ignores the varying degrees of influence that different dimensions exert on empowerment [24,33]. The absence of a nuanced approach to weighting each variable and dimension undermines the validity of such indices, leading to oversimplified conclusions that do not accurately reflect the lived realities of Indian women. Furthermore, traditional gender roles and expectations deeply ingrained in society often hamper the accurate assessment of women’s autonomy, agency, and empowerment within households and communities. This limited perspective hinders a comprehensive understanding of the complex processes and factors contributing to women’s empowerment in India. There is an unmet need for a dynamic assessment of the fluidity of women’s empowerment, reflecting its evolving nature. Hence, our study aims to explore the evolving nature of women’s empowerment in India, addressing both its multidimensional aspects and temporal changes from 2006 to 2021.

## Literature review

Women’s empowerment is a multidimensional construct encompassing decision-making, attitudes toward violence, mobility, financial security, perceived sexual rights, and societal norms [14,21,24,30–32,34–36]. Each dimension reflects different facets of women’s empowerment. This literature review synthesizes existing studies on the key dimensions of women’s empowerment, emphasizing their interconnected nature and critical role in fostering gender equality. Among all domains, decision-making is a foundational measure of empowerment that encompasses household decisions regarding healthcare, major purchases, visits to family, and financial matters [21,24]. Women who participate in these decisions tend to have improved access to healthcare and better child health outcomes [37]. However, studies indicated that in patriarchal societies, joint decision-making is more prevalent than sole female decision-making, suggesting that while progress has been made, full autonomy remains constrained [21,38]. Another study from South Asia highlights that even when women contribute economically, their ability to make independent decisions remains limited because of structural constraints [39].

Freedom of mobility represents an essential aspect of women’s empowerment as it reflects their ability to navigate public spaces without restrictions [1,30]. However, the interplay between mobility and other dimensions of empowerment is complex, because mobility alone does not necessarily translate into decision-making power [40,41].

Financial independence is another crucial driver of empowerment, and it is often measured through asset ownership, employment, and control over financial resources [42,43]. Women with bank accounts, land, or independent income sources exhibit higher autonomy in household decision-making [35,43,44]. However, employment alone does not ensure empowerment; control over earnings and financial literacy are equally critical [45]. Studies highlight the interaction between financial security and other empowerment domains such as mobility and decision-making, collectively enhance women’s empowerment [46]. Moreover, financial literacy significantly affects women’s ability to manage resources effectively, leading to increased bargaining power within households [47].

Further, perceived sexual rights reflect women’s autonomy in reproductive decisions and bodily integrity [25,26,48]. Studies have found that women who assert sexual rights experience lower rates of intimate partner violence (IPV) and better reproductive health outcomes [29,48,49]. However, social stigma and lack of awareness often limit women’s ability to exercise these rights, particularly in conservative societies [28,33]. Education and media exposure play crucial roles in enhancing women’s knowledge and confidence in asserting their sexual rights [12,32,43]. Furthermore, women who actively participate in reproductive decision-making are more likely to access maternal healthcare services and use contraception effectively [25,34].

Attitudes toward domestic violence also serve as a key indicator of women’s empowerment, reflecting the extent to which women internalize or reject norms that justify intimate partner violence (IPV) is correlated to their level of empowerment [17,29]. Rejection of violence in specific scenarios, such as going out without informing husband, neglecting children, arguing, refusing sex, or burning food, has been explored in multiple settings [24,28]. Furthermore, interventions that focus on shifting societal attitudes toward gender-based violence have a positive impact on empowerment [48–50].

Another key domain, societal norms, emphasizes the need to move beyond individual agency to address broader structural barriers. Societal norms represent the structural and cultural determinants of women’s empowerment [15]. This dimension encompasses education, media exposure, marital age, and gender-based disparities that influence long-term empowerment trajectories [21]. Since 2012, the SWPER index has incorporated media exposure, education, age at first birth, age at first cohabitation, spousal age difference, and educational disparity indicators, which provide a comprehensive framework for assessing societal influences on empowerment. Media exposure has been widely examined for its role in shaping attitudes toward gender equality and women’s autonomy [12,43]. Studies have demonstrated that women exposed to television, radio, and print media exhibit greater knowledge of reproductive rights and higher rejection of gender-based violence [24,43]. Education remains one of the most well-documented predictors of empowerment. Higher education levels correlate with greater decision-making power, economic independence, and reproductive autonomy [32,43]. However, disparities in access to quality education remain a significant challenge, particularly in rural areas [39,46]. Early marriage and teenage pregnancy further hinder women’s empowerment [41]. Studies have consistently found that delaying marriage and childbirth leads to improved health, education, and economic outcomes [32,41,51]. Social interventions aimed at delaying marriage have positive effects on female autonomy in decision-making and financial security [41,47]. The age and educational differences between spouses also reflect power dynamics within households [38,51]. Studies indicate that smaller age gaps and higher educational parity between spouses are associated with more egalitarian relationships and greater female empowerment [22,33,38]. However, patriarchal norms in many societies continue to prioritize male authority, limiting the transformative potential of these factors [52]. Research has highlighted the importance of legal reforms targeting child marriage and gender disparities in education as essential strategies for shifting societal norms and promoting empowerment [24,41].

Furthermore, women’s involvement in agriculture is crucial for economic growth and food security. Studies have shown that land ownership significantly enhances women’s bargaining power within households, leading to improved agricultural productivity and household welfare [33,42]. Although women comprise a substantial share of the agricultural workforce, they face constraints in accessing land, credit, and extension services [53]. Research indicates that women’s control over agricultural resources is linked to better child nutrition and food security [42,47]. Policies promoting joint land ownership and women-focused agricultural programs have had positive impacts in several developing countries [33,42,47]. Another dimension that emerges as one of the important indicators of women’s participation in the political aspect of the country. This reflects women’s ability to influence governance and decision-making structures [54,55]. Quotas and reservations for women in political offices have increased female representation and improved policy focus on gender-sensitive issues [54]. Studies have found that women in leadership roles often prioritize issues such as education, health, and gender-based violence [55]. However, social norms, limited political networks, and male dominance continue to restrict women’s political participation [56,57]. Strengthening women’s civic engagement and political literacy remains key to achieving equitable governance [58,59]. Overall, the literature underscores that women’s empowerment is a dynamic, multifaceted process influenced by intersecting socio-cultural, economic, and political factors. Each domain plays a critical role in shaping women’s autonomy agency, and empowerment; however, their impact is amplified when considered together. In this study, women’s empowerment is operationalized as a six dimensional construct: decision-making, attitude toward violence, perceived sexual rights, financial independence, freedom of mobility, and social independence.

## Materials and methods

### Data source

The study used data from the third (2005−06), fourth round (2015−16) and fifth (2019−21) rounds of the National Family Health Survey (NFHS) [60–62], a large-scale, multi-round survey conducted on a nationally representative sample of households in India. The NFHS provides consistent and reliable estimates of fertility, mortality, family planning, maternal and child health care utilization, nutritional status, anaemia, HIV/AIDS, and women’s sexual and reproductive health at the national, state, and district levels (from NFHS-4 onwards).

NFHS-3 used the 2001 Census as its sampling frame and adopted a multi-stage stratified sampling design to ensure representativeness at national, state, and urban/rural levels across 29 states and union territories. Rural areas followed a two-stage design where villages (primary sampling units, or PSUs) were selected using Probability Proportional to Size (PPS), and households were chosen through systematic random sampling. Urban areas followed a three-stage design, with wards selected by PPS, one Census Enumeration Block (CEB) randomly chosen from each ward, and households systematically selected within each CEB. NFHS-3 collected data from 109,041 households, 124,385 women aged 15–49, and 74,369 men aged 15–54, including information on women’s participation in household decision-making, mobility, earnings, and bank account ownership. In NFHS-4 and NFHS-5, two additional indicators, namely ownership of mobile phones and ownership of house or land, have been included. The NFHS-4 sample is a stratified two-stage sample. The 2011 census served as the sampling frame for the selection of PSUs. PSUs were villages in rural areas and Census Enumeration Blocks (CEBs) in urban areas. PSUs with fewer than 40 households were linked to the nearest PSU. Within each rural stratum, villages were selected from the sampling frame with probability proportional to size (PPS). Where the women’s empowerment variables are collected at the state level. A subsample of 15 percent of households was selected for the implementation of the state module. In the 15 percent of selected households, a long questionnaire was administered that included all the questions needed for district-level estimates plus additional questions for women’s empowerment. To achieve a representative sample of 15 percent of households, NFHS-4 conducted interviews in every alternately selected household in 30 percent of the selected clusters. The same sampling frame and design was used for NFHS-5. NFHS-4 covered 699,686 women from over 601,000 households across 640 districts. NFHS-5 fieldwork for India was conducted in two phases: phase one from 17 June 2019 to January 30, 2020, and phase two from January 2, 2020, to April 30, 2021. Information was gathered from 636,699 households, 724,115 women, and 101,839 men.

For our study, we have used a women’s file with a sample size of 87,925 in NFHS-3, 86,811 in NFHS-4, and 76,910 in NFHS-5 at the state level.

### Variable description

#### Dependent variable.

27 items were taken to construct the index of women’s empowerment under six dimensions:

- *Attitude towards Violence*: Respondents answered questions on whether beating is justified if the wife goes out without telling the husband, neglects children, argues, refuses sex, or burns food. “1” if not justified, otherwise “0.”
- *Decision-Making*: Assessed by who decides on healthcare, large purchases, family visits, and money management. “1” if the respondent decides, “0” if not.
- *Perceived sexual rights*: Respondents justified refusal of sexual intercourse if the husband had other partners, STDs, or the wife did not want to have sexual intercourse. “1” if justified, otherwise “0.”
- *Freedom of Mobility*: Assessed permission to visit the market, health facilities, and village alone or with someone else. “1” if allowed, “0” if restricted.
- *Financial Independence*: Questions on working in the last 12 months (“1” If yes, otherwise “0”), owning a bank account (“1” If yes, otherwise “0”), owning land (“1” If yes, otherwise “0”), owning a household (“1” If yes, otherwise “0”), having a mobile phone (“1” If yes, otherwise “0”), and having any money of your own that you alone can decide how to use (“1” If yes, otherwise “0”).
- *Societal Norms*: Mass media exposure (If the respondent is not at all exposed to any of the mass media, which are reading the newspapers, listening to radio and watching Television, coded as “0”; otherwise “, 1”). Women’s education (secondary and higher education coded as “1” and “0” otherwise). Age of respondent at cohabitation (18 and above coded as “1” and “0” otherwise). Age of respondent at first birth (18 and above coded as “1” and “0” otherwise). Age difference (same age or above coded as “1” and “0” otherwise). Education difference (same or higher education coded as “1” and “0” otherwise

#### Independent variables.

The independent variables used in this study are age of the respondent (15–19 years, 20–24 years, 25–29 years, 30–34 years, 35–39 years, 40–44 years, 45–49 years), occupation of the respondent (Not in the Workforce, Agriculture, Others), NFHS does not provide the “income of the individual”; hence, “wealth quintile” has been used as a proxy (Poorest, Poorer, Middle, Richer, Richest), place of residence (Rural, Urban), religion (Hindu, Muslim, Others), caste (Scheduled Caste, Scheduled Tribe, Other Backward Classes, Others), region (North, Central, East, Northeast, West, South)

### Statistical methodology

In this study, we have used second-order Confirmatory Factor Analysis (CFA) to construct the women’s empowerment index. CFA is a theory-driven multivariate statistical technique used to test hypotheses about the structure of latent variables based on prior theory by specifying the number of factors and the pattern of indicator-factor relationships. Traditionally, before using CFA, researchers use exploratory factor analysis (EFA). The motive behind using the EFA is to identify the number of latent variables and underlying factor structure. However, in our study, we have pre-defined dimensions and variables which have been pre-tested in previous studies [15–17,22,24,63]. CFA aims to reproduce the sample variance-covariance matrix by the parameter estimates of the measurement solution. It indicates the structure a priori specifying zero factor loading to items that do not belong to the factor and those that belong to the scale with nonzero factor loadings [64].

#### Model specification.

**Fig 1:**
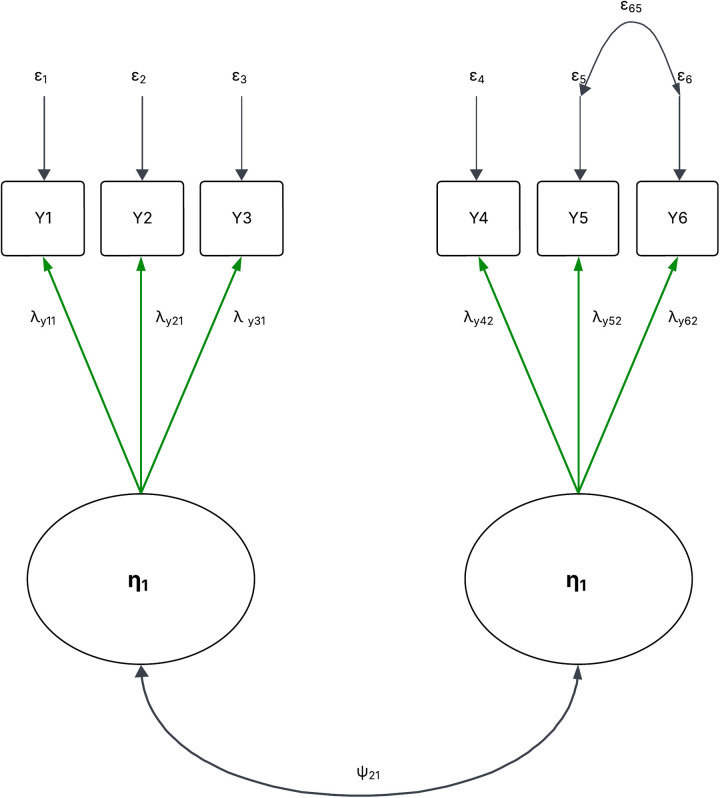
Covariance structure in CFA.


CFA is based on the common factor model, which assumes that each observed variable is a linear combination of one or more latent factors and an error term (Fig 1).

$$Y=\Lambda_{y}\eta +\;\epsilon$$

Where, Y is a n × 1 vector of observed indicators ($Y_{1},\;Y_{2},\;Y_{3},\ldots ,Y_{n}$)

$\Lambda_{y}$ is a n × 2 factor loading matrix containing the loading (λ) that measure the strength of the relationships between latent constructs and observed indicators.

ϵ is a p × 1 vector of measurement error

The ($Y_{1\ldots n}$), measure loads on the first exogenous factors ($\xi_{1\ldots n}$), and $\lambda_{Y21}$ indicate Y2 also loads on ($\xi_{1}$) and Ψ is factor variance and covariance. The predicted covariance of two indicators that load on the same factor is computed as the product of their factor loadings, the variance of the factor. The model-implied covariance of two indicators that loads on separate factors is estimated as the product of their factor loadings times the factor covariance. The following equation can reproduce the variance of X2


$$Var(Y2)=\sigma_{22}=\lambda Y212\phi_{11}+\delta_{2}$$

The squared loading represents the proportion of variance in the indicator that is explained by the factor (communality). The commonality of Y2 is


$$\eta_{22}^{2}=\lambda Y212$$

Residual variances can be readily calculated as


$$\delta_{2}=1-\lambda Y212$$

The predicted covariance between Y2 and Y3 is estimated as follows


$$Cov(Y2,\;Y3)=\sigma_{3,2}=\;\lambda Y21\;\phi_{11}\lambda Y31\;$$

The predicted covariance between Y3 and Y4 (indicators that load on separate factors) is estimated as follows


$$Cov(Y2,\;Y4)=\sigma_{4,3}=\;\lambda Y31\;\phi_{31}\lambda Y41\;$$

#### Model estimation.

CFA parameters are estimated using Maximum Liklihood Estimation (MLE) by minimizing the discrepancy between the observed and model-implied covariance matrices:


$$ℒ(\theta )=-\frac{n}{2}\left[Log\left|\Sigma (\theta )\right|+tr(S\Sigma^{-1}(\theta ))\right]$$

Where,

n is the sample size

S is the sample covariance matrix.

(𝜃) is the model-implied covariance matrix

After creating a single score for the empowerment of every woman, we have divided it into three parts based on the tercile. Women who fall under the lowest tercile are considered to have low empowerment; the middle tercile is coded as middle/partial empowerment, and women whose empowerment score falls in the highest tercile are known as the highly empowered women.

Further, we used descriptive analysis, multinomial logistic regression and Age-Period-Cohort (APC) analysis.

Multinomial logistic regression predicts the probability of the categorical outcome based on the given independent variables. These independent variables can be dichotomous, categorical or continuous (interval or ratio in scale). It is an extended form of the binary logistic regression which considers more than two categories of the outcome variables. Multinomial logistic regression uses MLE similarly as binary logistic regression to calculate the relative risk of the categorical outcome. The relative risk for one unit change in $x_{i}$ is


$$e^{\beta_{i}^{\left(2\right)}}=\;\frac{e^{\beta_{1}^{\left(2\right)}x_{1}+\ldots +\beta_{i}^{\left(2\right)}{(x}_{i}+1)+\ldots +\beta_{k}^{\left(2\right)}x_{k}}}{e^{\beta_{1}^{\left(2\right)}x_{1}+\ldots +\beta_{i}^{\left(2\right)}x_{i}+\ldots +\beta_{k}^{\left(2\right)}x_{k}}}$$

Where X and $\beta_{k}^{\left(2\right)}$ are vectors equal to ($x_{1},x_{2},\ldots ,x_{k}$) and ($\beta_{1}^{\left(2\right)},\;\beta_{2}^{\left(2\right)},\ldots ,\;\beta_{k}^{\left(2\right)}$), respectively.

For APC analysis, in single-year increments, cohort and age were evaluated. A major challenge in APC analysis is the identification problem, which arises from the perfect linear dependency among age, period, and cohort effects. Several approaches have been developed to address this issue, including Yang model [65], the Hanoch-Honig/Deaton-Paxson normalisation approach [66,67], and the Maximum Entropy (ME) approach put out by Browning, Crawford, and Knoef [66]. While, the Yang model is commonly used in epidemiological and demographic studies. Nevertheless, this approach has drawbacks, including obtaining data from many time periods and placing restrictions on the geometric orientation of parameter vectors [66]. Similarly, the Deaton-Paxson normalization approach, though methodologically robust, can lead to biased estimates when the assumptions about linear trends in cohort effects are not met [68]. Given these considerations, the ME technique was chosen for this study due to its flexibility in resolving identification issues without imposing rigid assumptions on functional forms. Unlike traditional approaches that estimate a unique solution based on strong parametric assumptions, the ME method determines the most probable solution by incorporating a probabilistic framework. This feature is particularly advantageous in situations where data are subject to measurement error or where structural changes across time may lead to violations of standard APC assumptions. Additionally, the ME approach is well-suited for bounded outcome variables (0,1), as it parametrizes APC model coefficients as probability distributions rather than fixed estimates, allowing for a more nuanced interpretation of cohort and period effects [66].

## Results

**Fig 2:**
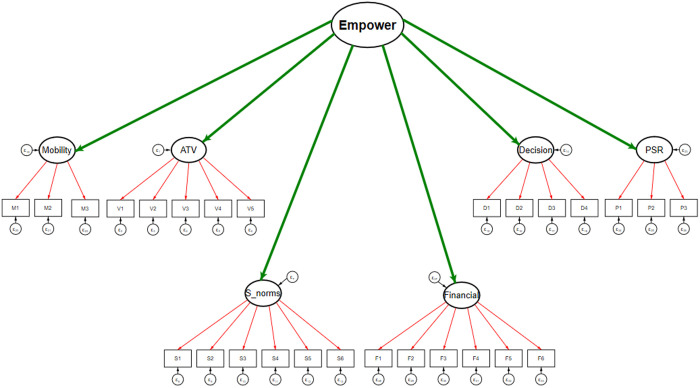
Path diagram used for constructing the 6-dimension model using second-order Confirmatory Factor Analysis, 2005−06, 2015−16 and 2019−21, India.

Before proceeding towards the construction of the women’s empowerment index, we have run a sample adequacy test to check if the extracted data is adequate enough to run further analysis, including Exploratory Factor Analysis (EFA) and Cronbach’s alpha to check the validity of each constructed domain for all the survey years (S1 File). Fig 2 shows the path diagram of the construction of the women’s empowerment index, and Table 1 presents the factor loadings of the variables and dimensions of the index. We used the second-order CFA model for all three survey rounds. The first-order model identified the standard factor loadings of the variables for the six broad dimensions of women’s empowerment, and the second-order model aggregated these dimensions into an overall empowerment index for women. The six primary dimensions are decision-making, attitude towards violence, freedom of mobility, perceived sexual rights, financial independence, and societal norms.

**Table 1:** Factor loadings of variables used in constructing the women’s empowerment index using Confirmatory Factor Analysis, 2005−06, 2015−16 and 2019−21, India.

Variables	Factor Loading		
	2005−06	2015−16	2019−21
**Attitude towards Violence (ATV)**			
V1: Not justified if wife goes out without telling husband	0.64*** [0.61, 0.67]	0.69*** [0.67, 0.70]	0.67*** [0.65, 0.68]
V2: Not justified if wife neglects the children	0.51*** [0.48, 0.54]	0.74*** [0.73, 0.75]	0.70*** [0.69, 0.71]
V3: Not justified if wife argues with husband	0.76*** [0.74, 0.78]	0.82*** [0.81, 0.83]	0.78*** [0.77, 0.79]
V4: Not justified if wife refuses to have sex with husband	0.69*** [0.66, 0.72]	0.59*** [0.58, 0.60]	0.61*** [0.60, 0.62]
V5: not justified if wife burns the food	0.68*** [0.67, 0.70]	0.64*** [0.63, 0.66]	0.61*** [0.60, 0.62]
**Decision-Making**			
D1: Decides on the respondent’s healthcare	0.61*** [0.60, 0.62]	0.76*** [0.74, 0.77]	0.75*** [0.75, 0.80]
D2: Decides on large household purchases	0.76*** [0.75, 0.77]	0.81*** [0.79, 0.83]	0.77*** [0.72, 0.77]
D3: Decides on visits to family or relatives	0.72*** [0.71, 0.72]	0.80*** [0.78, 0.82]	0.78*** [0.75, 0.80]
D4: Deciding what to do with money husband earns	0.58*** [0.56, 0.59]	0.73*** [0.72, 0.75]	0.76*** [0.73, 0.78]
**Freedom of Mobility**			
M1: Go to the market	0.93*** [0.93, 0.94]	0.91*** [0.90, 0.92]	0.75*** [0.73, 0.76]
M2: Go to the health facility	0.86*** [0.85, 0.87]	0.87*** [0.85, 0.88]	0.90*** [0.89, 0.91]
M3: Go to the village	0.72*** [0.70, 0.74]	0.80*** [0.77, 0.82]	0.77*** [0.76, 0.79]
**Perceived Sexual Rights**			
P1: If a wife knows her husband has sex with other women	0.77*** [0.76, 0.77]	0.85*** [0.85, 0.86]	0.75*** [0.74, 0.76]
P2: Say no to your husband if you do not want to have sexual intercourse.	0.86*** [0.85, 0.87]	0.92*** [0.92, 0.93]	0.83*** [0.82, 0.84]
P3: If a wife knows her husband has a sexually transmitted disease	0.74*** [0.73, 0.80]	0.78*** [0.77, 0.79]	0.61*** [0.60, 0.62]
**Financial Independence**			
F1: Have a bank account	–	0.53*** [0.51, 0.54]	0.02* [0.01, 0.03]
F2: Own a house	–	0.04* [0.03, 0.07]	0.87*** [0.82, 0.90]
F3: Have a mobile phone	–	0.55*** [0.54, 0.57]	0.60*** [0.58, 0.63]
F4: Own a land	–	0.07*** [0.05, 0.08]	0.85*** [0.81, 0.89]
F5: Working in the last 12 months	–	0.14*** [0.12, 0.16]	0.25** [0.23, 0.27]
F6: do you have any money of your own that you alone can decide how to use	–	0.25*** [0.20, 0.29]	0.42*** [0.39, 0.45]
**Societal Norms**			
S1: Awareness using Media exposure (read, radio and TV).	0.35*** [0.34, 0.37]	0.33*** [0.30, 0.36]	0.38*** [0.36, 0.41]
S2: Women’s education.	0.78*** [0.77, 0.80]	0.66*** [0.59, 0.73]	0.89*** [0.81, 0.97]
S3: Age of respondent at cohabitation.	0.62*** [0.61, 0.63]	0.45*** [0.42, 0.49]	0.44*** [0.40, 0.48]
S4: Age of respondent at first birth.	0.49*** [0.47, 0.50]	0.29*** [0.26, 0.33]	0.38*** [0.36, 0.4]
S5: Age difference: woman’s minus husband’s age.	0.01 [0.01, 0.03]	0.03** [0.01, 0.05]	0.35*** [0.33, 0.37]
S6: Education difference: woman’s minus husband’s years of schooling.	0.21*** [0.20, 0.22]	0.29*** [0.27, 0.31]	0.30*** [0.27, 0.33]
**Empowerment**			
Attitude towards Violence	0.26*** [0.23, 0.30]	0.29*** [0.25, 0.32]	0.41*** [0.38, 0.53]
Decision-Making	0.32*** [0.29, 0.37]	0.21*** [0.18, 0.23]	0.22** [0.19, 0.25]
Freedom of Mobility	0.47*** [0.43, 0.52]	0.11*** [0.08, 0.13]	0.12*** [0.10, 0.14]
Perceived Sexual Right	0.16*** [0.14, 0.17]	0.18*** [0.14, 0.21]	0.40*** [0.35, 0.44]
Financial Independence	–	0.44*** [0.28, 0.59]	0.10*** [0.01, 0.07]
Societal Norms	0.34*** [0.31, 0.37]	0.37*** [0.33, 0.41]	0.21** [0.18, 0.23]

* p < 0.05, ** p < 0.01, *** p < 0.001

Table 1 presents the factor loadings of variables used in constructing the women’s empowerment index for the years 2005−06, 2015−16 and 2019−21. Variable D2 (household purchase) and D3 (visits to family/relatives) emerged as crucial factors in the construction of the decision-making dimension across all survey years (coef.>=0.72). However, 2015−16 and 2019−21 showed increased loading for all decision-making variables compared to 2005−06, indicating a trend towards greater empowerment in household decision-making over time. Variable V3 (argues with husband) emerged as a critical variable in shaping “Attitude towards Violence” and exhibits a significant standard factor loading above 0.76 for all the survey years. However, there was a drop observed in the factor loadings of variables V4 (refuse sex) and V5 (burn food) in 2015−16, which signifies fluctuations in the norms regarding women’s attitudes towards violence. Freedom of mobility demonstrated a strong loading of variables M1 (market access) in 2005−06 and 2015−16, but a decline from 0.93 in 2005−06 to 0.75 in 2019−21, and M2 became the strong indicator with the loading of 0.90 indicating a possible restriction and shift in women’s freedom over time. Perceived sexual rights showed variability, with P1 (refusal if the husband had other partners) peaking in 2015−16 (loading = 0.85) but dropped to 0.75 in 2019−21, reflecting changing perceptions of sexual autonomy. In 2005−06, ownership of mobile phone, house and land were not recorded in the data, and remaining variables related to the “Financial Independence” dimension were insignificant, showing a complete absence of financial empowerment. However, from 2015−16, there has been a significant increase, particularly in F2 (owning household) and F4 (owning land), highlighting the importance of property ownership in women’s empowerment.

The second-order CFA model amalgamated these first-order factors into a comprehensive border dimension of women’s empowerment, leading to a single construct. Attitude towards violence consistently illustrated a strong involvement in constructing the index in all survey years. Decision-making, although it depicted a slight decrease from 2005−06 to 2019−21, remained a significant dimension of women's empowerment. Further, we did not have to construct the “Financial Independence” dimension because of the lack of sufficient variables during 2005−06. However, by 2015−16, it was an essential dimension. Furthermore, societal norms showed a substantial increase from 2005−06 to 2019−21, underscoring the growing impact of social norms on empowerment.

Table 2 presents the validity of the index by providing goodness-of-fit indices for 2005–06, 2015–16 and 2019–2021 of the second-order CFA. The RMSEA value in all survey years was below the cutoff point, which was 0.05. The CFI and TLI values ranged from 0.960 to 0.998, which was higher than 0.95, indicating strong reliability. Finally, the SRMR value was less than 0.08 for all survey years. These results validate the robustness and reliability of the women’s empowerment index across all three NFHS surveys. Further, fit indices of the first-order CFA for all the survey years are given in the supplementary file (S2 File).

**Table 2:** Construct validity of the women’s empowerment index obtained from second-order Confirmatory Factor Analysis, 2005–06, 2015–16 and 2019–2021, India.

Survey	RMSEA	CFI	TLI	SRMR
2005−06	0.005	0.981	0.995	0.003
2015−16	0.010	0.984	0.979	0.004
2019−21	0.007	0.997	0.977	0.002

RMSEA: Root mean squared error of approximation, CFI: Comparative fit index, TLI: Tucker–Lewis index, SRMR: Standardized root mean squared residual

Table 3 presents the prevalence of women’s empowerment by their background variables from 2006 to 2021. It reveals that the prevalence of highly empowered women is increasing as their age increases during NFHS-3. However, by NFHS-4 and NFHS-5, the higher prevalence of empowered women lies in the younger age groups, especially among women aged 30–40 years. Meanwhile, women involved in the agriculture sector have the lowest prevalence of high empowerment across all the survey years. The wealth quintile showed an increase in the prevalence of high empowerment in all the quintiles from 2006 to 2021. However, the lowest wealth quintile depicts a lower prevalence of highly empowered women than the women who belong to the higher wealth quintile in all the survey years. Muslim women and women who reside in rural areas have a low prevalence of high empowerment. The central region of India had the lowest prevalence of high empowered women during NFHS-3. However, it showed an increase of eight percent point from 2005−06 to 2019−21. The Northern region showed a notable increase in high empowerment from twenty-eight percent in 2015−16 to forty-four in 2019−21. The southern region had highest prevalence of high empowerment in all the survey years.

**Table 3:** Prevalence of women’s empowerment by the background variables, 2005-06, 2015-16 and 2019-2021, India.

Background Variables	2005−06				2015−16				2015−16			
Women’s Empowerment	Low (%)	Medium (%)	High (%)	Total (Freq)	Low (%)	Medium (%)	High (%)	Total (Freq)	Low (%)	Medium (%)	High (%)	Total (Freq)
Mother’s Age												
15–19	68.2	24.3	7.4	6,353	47.5	32.9	19.6	3,026	40.8	36.4	22.7	2,274
20–24	46.3	33.6	20.1	15,851	33.7	35.7	30.7	13,429	32.8	34.6	32.6	10,649
25–29	32.2	35.3	32.6	17,512	29.1	33.9	37	17,363	31.8	31.7	36.6	15,024
30–34	26.9	33.4	39.7	15,546	29.9	31.9	38.2	15,748	31.6	31.8	36.6	14,099
35–39	24.9	33.6	41.5	13,688	32.7	31.2	36	14,148	33.3	32.8	33.9	13,344
40–44	23.7	34.2	42.1	10,961	35.7	33.3	31	12,033	34.2	34.8	31	10,724
45–49	22.8	33.8	43.4	8,014	38.9	34.4	26.7	11,064	36	34.9	29.1	10,796
Occupation												
Not in the Workforce	33.4	32.8	33.8	50,332	33.2	33.1	33.7	60,248	32	32.9	35.1	52,347
Agricultural	40.7	36.3	23	23,875	44.8	37.9	17.3	13,765	41.8	36.7	21.6	12,056
Other	25.3	40.1	45.7	13,648	22.2	30.2	47.6	11,870	18.9	31.9	49.2	12,506
Wealth Quintile												
Poorest	46.9	34.3	18.7	16,458	54.7	34.3	11	14,293	41.5	36.7	21.8	14,419
Poorer	43.9	35.1	21	17,469	45.8	36.1	18.1	16,513	38.6	35.1	26.3	15,662
Middle	37.4	36.1	26.5	17,635	35.9	37.3	26.8	17,986	36	34.2	29.8	15,800
Richer	28.4	34.6	37	17,932	25.1	34.7	40.2	18,659	30.1	33.6	36.3	15,700
Richest	22.1	26.8	51	18,431	12.6	25.2	62.3	19,361	10.7	22.3	67	15,328
Community												
SC/ST	35.8	36	28.2	23,298	40.5	34.1	25.3	24,339	35.4	34.8	29.8	23,597
OBC	36.3	33.5	30.2	34,660	33.2	34.9	31.9	38,362	34.3	34.9	30.8	33,775
Others	26.9	30.8	42.3	26,682	24.6	29.4	45.9	20,200	27.1	29.3	43.6	15,889
Religion												
Hindu	33.2	33.4	33.4	71,594	33.6	33.1	33.2	70,561	32.8	33.4	33.7	62,456
Muslim	41	33.6	25.5	11,607	36.2	35.5	28.3	11,778	38.2	33.4	28.4	10,585
Others	19.4	31.5	49.1	4,724	21	31.1	47.9	4,472	20.9	31.9	47.2	3,869
Place of Residence												
Urban	20.2	30.4	49.4	27,018	21.7	30.6	47.7	30,099	25.6	30.7	43.7	23,821
Rural	40.5	34.6	24.9	60,907	39.5	34.8	25.7	56,712	36.8	34.5	28.7	53,089
Region												
North	36	35.9	28	7,638	24.2	30.1	45.7	11,457	25.1	30.7	44.2	10,799
Central	42	32.3	25.8	21,722	35.7	35.3	29	17,939	31.4	34.8	33.7	17,782
East	30	31.7	38.3	12,276	41.3	33.4	25.3	18,680	35	33.3	31.6	18,565
Northeast	18.8	37.2	44.1	3,311	31.4	32.6	36	2,787	28.3	30.6	41.1	2,866
West	30.8	33.9	35.3	20,360	30	33.3	36.7	14,281	26.9	30.2	42.9	10,874
South	22.9	31.8	45.3	13,645	20.7	33.5	45.8	21,667	19.6	36.1	44.3	16,024
Total				87,925				86,811				76,910

The results of multinomial logistic regression analysis indicate significant changes in the factors influencing women’s empowerment in India where low empowerment serves as the base outcome. Age is strongly associated with middle and high empowerment during NFHS-3. Women aged 45−49 were ten times more likely to be high empowered compared to women aged 15–19. However, by NFHS-4, women in older age groups no longer demonstrate a significant association with being empowered. In NFHS-4 and NFHS-5, there was a noticeable shift in the trend towards younger women. Women aged 30–39 are more likely to exhibit higher levels of empowerment (Table 4).

**Table 4:** Multinomial logistic regression of women’s empowerment by background variables, NFHS-3, NFHS-4, and NFHS-5, India.

Women’s Empowerment	NFHS-3		NFHS-4		NFHS-5	
	Middle Empowerment	Higher Empowerment	Middle Empowerment	Higher Empowerment	Middle Empowerment	Higher Empowerment
Mother’s Age						
15-19 ®						
20-24	1.82*** [1.64,2.02]	3.14*** [2.68,3.68]	1.42*** [1.24,1.63]	1.73*** [1.43,2.09]	1.12 [0.96,1.31]	1.55*** [1.30,1.84]
25-29	2.67*** [2.40,2.96]	6.45*** [5.53,7.53]	1.51*** [1.32,1.73]	2.11*** [1.75,2.54]	1.07 [0.92,1.24]	1.70*** [1.43,2.02]
30-34	3.00*** [2.70,3.35]	9.31*** [7.96,10.88]	1.35*** [1.18,1.54]	1.90*** [1.58,2.30]	1.05 [0.90,1.22]	1.63*** [1.37,1.94]
35-39	3.23*** [2.89,3.61]	9.83*** [8.39,11.52]	1.17* [1.02,1.34]	1.57*** [1.29,1.89]	1.04 [0.89,1.20]	1.43*** [1.20,1.70]
40-44	3.21*** [2.86,3.61]	9.74*** [8.29,11.45]	1.1 [0.96,1.27]	1.07 [0.88,1.29]	1.05 [0.90,1.22]	1.21* [1.01,1.44]
45-49	3.31*** [2.92,3.75]	10.35*** [8.75,12.25]	1.02 [0.89,1.17]	0.83 [0.68,1.01]	1.01 [0.87,1.18]	1.1 [0.92,1.31]
Occupation						
Not in the Workforce ®						
Agricultural	1.05 [0.99,1.12]	1.03 [0.96,1.11]	1.11*** [1.05,1.18]	0.86*** [0.79,0.93]	0.97 [0.91,1.04]	0.97*** [0.92,1.03]
Other	1.36*** [1.26,1.47]	2.19*** [2.03,2.36]	1.47*** [1.35,1.60]	2.65*** [2.42,2.89]	1.03 [0.95,1.11]	3.15*** [3.06,3.24]
Wealth Quintile						
Poorest ®						
Poorer	1.10* [1.02,1.19]	1.16** [1.06,1.28]	1.27*** [1.19,1.36]	2.01*** [1.83,2.20]	1.86 [0.99,1.94]	3.37*** [3.27,3.48]
Middle	1.31*** [1.21,1.41]	1.57*** [1.43,1.72]	1.70*** [1.58,1.83]	3.82*** [3.48,4.20]	1.97*** [1.09,2.27]	4.92*** [4.76,5.09]
Richer	1.47*** [1.35,1.61]	2.37*** [2.15,2.61]	2.26*** [2.07,2.46]	5.16*** [4.01,6.04]	2.37*** [2.25,2.49]	7.74*** [7.50,8.00]
Richest	2.32*** [2.09,2.58]	6.49*** [5.81,7.25]	3.24*** [2.89,3.64]	7.88*** [7.10,8.02]	4.51*** [4.36,4.68]	9.76*** [9.28,10.30]
Community						
SC/ST ®						
OBC	0.90*** [0.85,0.96]	0.96 [0.90,1.03]	1.13*** [1.07,1.20]	1.16*** [1.08,1.24]	1.03 [0.97,1.09]	1.01 [0.95,1.08]
Others	0.93* [0.86,0.99]	1.18*** [1.09,1.26]	1.14** [1.05,1.23]	1.58*** [1.45,1.72]	0.98 [0.91,1.06]	1.22*** [1.12,1.32]
Religion						
Hindu ®						
Muslim	0.77*** [0.71,0.84]	0.51*** [0.47,0.56]	0.91* [0.85,0.99]	0.60*** [0.55,0.65]	0.83*** [0.76,0.90]	0.61*** [0.55,0.66]
Others	1.39*** [1.25,1.55]	1.76*** [1.58,1.96]	1.40*** [1.24,1.59]	1.68*** [1.48,1.90]	1.07 [0.95,1.20]	1.07 [0.96,1.20]
Place of Residence						
Urban ®						
Rural	0.43*** [0.39,0.49]	0.49*** [0.46,0.52]	0.52* [0.56,0.59]	0.66*** [0.60,0.72]	.65*** [0.69,0.72]	0.73*** [0.68,0.79]
Region						
North ®						
Central	1.17*** [1.07,1.28]	1.07 [0.98,1.18]	1.03 [0.96,1.11]	0.91* [0.84,0.98]	1.01 [0.94,1.09]	0.94 [0.88,1.02]
East	1.02 [0.93,1.08]	0.97 [.89,1.05]	0.93 [0.86,1.00]	0.88** [0.81,0.96]	0.96 [0.89,1.04]	0.99 [0.91,1.08]
Northeast	2.52*** [2.26,2.82]	3.67*** [3.28,4.10]	1.22*** [1.10,1.34]	1.60*** [1.44,1.77]	1.06 [0.95,1.18]	1.73*** [1.56,1.91]
West	0.33*** [0.22,0.45]	1.56*** [1.43,1.70]	0.87** [0.79,0.96]	0.64*** [0.58,0.71]	0.95 [0.86,1.05]	1.03 [0.94,1.14]
South	1.06 [0.99,1.14]	1.01 [0.93,1.08]	0.78 [0.72,0.85]	0.59 [0.54,0.64]	0.65 [0.60,0.70]	0.27 [0.24,0.29]

* p < 0.05, ** p < 0.01, *** p < 0.001; Reference category: Low empowerment

Women engaged in non-agricultural employment consistently show higher relative risks of high empowerment. In NFHS-3, those in ‘Other’ occupations (non-agricultural work) were 2.19 times more likely to be highly empowered than those not in the workforce. This advantage becomes more pronounced in NFHS-5, where the relative risk (RRR) increases to 3.15 for high empowerment. Wealth status remains one of the strongest and most consistent predictors of empowerment across all rounds. Compared to the poorest women, women in the richest quintile had higher chances of being empowered. The RRRs for high empowerment increased from 6.49 in NFHS-3 to 9.76 in NFHS-5. In NFHS-3, women from ‘Other’ caste groups (neither SC/ST nor OBC) had higher relative risks of high empowerment (RRR = 1.18), and this advantage grows in NFHS-4 (RRR = 1.58) and NFHS-5 (RRR = 1.22). Conversely, Muslim women remain consistently disadvantaged compared to Hindu women, across all rounds. Urban-rural differences in empowerment persisted in all the survey years. In NFHS-3, rural women had a 51% lower chance of being highly empowered. Though this gap narrowed slightly in subsequent rounds, it remained substantial in NFHS-5 (RRR = 0.73).

**Fig 3:**
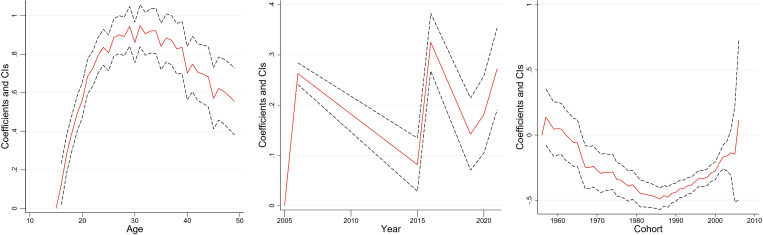
Age, period, and cohort effects on women’s empowerment, India, 2005–06, 2015–16 and 2019–2021.

**Fig 4:**
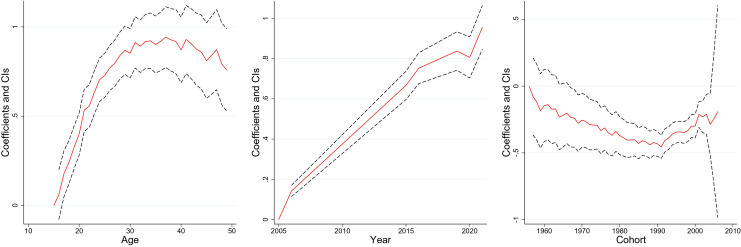
Age, period, and cohort effects on “decision–making” dimension, India, 2005–06, 2015–16 and 2019–2021.

**Fig 5:**
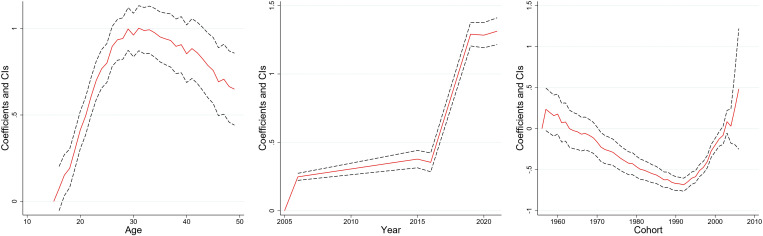
Age, period, and cohort effects on “freedom of mobility” dimension, India, 2005–06, 2015–16 and 2019–2021.

**Fig 6:**
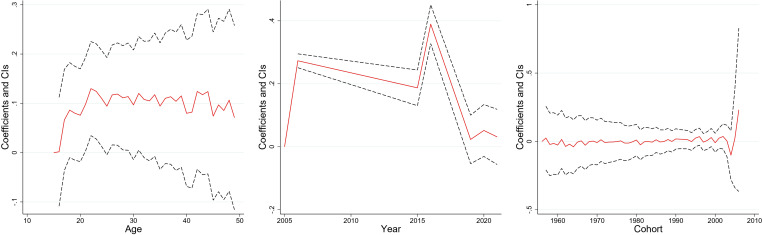
Age, period, and cohort effects on “perceived sexual rights” dimension, India, 2005–06, 2015–16 and 2019–2021.

**Fig 7:**
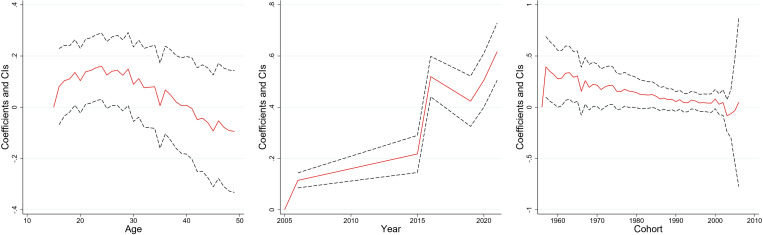
Age, period, and cohort effects on “attitude toward violence” dimension, India, 2005–06, 2015–16 and 2019–2021.

**Fig 8:**
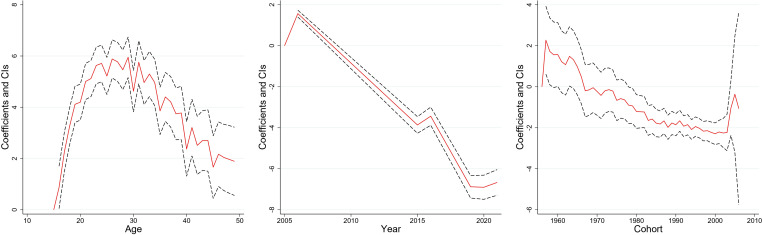
Age, period, and cohort effects on “societal norms” dimension, India, 2005–06, 2015–16 and 2019–2021.

Figs 3–8 and S3 Table (supplementary S3 File) illustrate the APC analysis findings. The findings demonstrated that age significantly impacted women’s empowerment and the other five dimensions of empowerment.

### Age effect

The degree of women’s empowerment consistently increased with age, attaining its peak between 25–35 years (Fig 3), followed by a gradual decline. This pattern remained evident in decision-making (Fig 4), and freedom of mobility (Fig 5) dimensions. Women attain their highest decision-making ability near the age of thirty, after which the effect becomes stagnant. Similarly, there was a substantial increase in the freedom of mobility until the early 30s, after which there was a slight decline. Further, Fig 8 shows that women aged 25–30 were more likely to internalize societal norms, reflecting their higher engagement in societal roles during this life stage. In contrast to the other dimensions, age had no significant effect on women’s perceived sexual rights (S3 File).

### Period effect

The period effect highlights significant temporal change in women’s empowerment across the three survey periods. A steep rise in empowerment was observed between 2005−06 and 2015−16, peaking in 2015−16 (Fig 3). However, a dip was observed between 2015−16 and 2019−2021. The decision-making dimension has had a steady, linear increase from 2005−06 to 2019−21 (Fig 4). Further, freedom of mobility showed a stagnant effect from 2005−06 to 2015−16 (Fig 5). Subsequently, there was a substantial increase between 2015−06 and 2019−21. Furthermore, only period had a significant effect on attitudes towards violence among women (Fig 7), with resistance to violence increasing over time (S3 File).

### Cohort effect

The cohort analysis revealed nuanced generational differences in women’s empowerment. Women born between 1957 and 1967 exhibited no significant cohort effect (S3 File). In contrast, women from the 1980–1990 cohort had lower empowerment level which started to escalate after the cohort of the early 2000s.

The cohort of 1957–1970 had an insignificant effect on the decision-making of women (S3 File). However, the younger cohorts demonstrated consistent improvement. Similarly, the cohort from 1957 to 1971 showed no significant effect on freedom of mobility, but notable variations emerged in later cohorts, with women born after 1990 exhibiting increased mobility compared to older cohorts (S3 File). Further, no significant cohort effects were observed for attitudes towards violence or perceived sexual rights, underscoring the persistent challenges in achieving autonomy in these dimensions across generations (S3 File).

## Discussion

The study conceptualizes women’s empowerment as a composite of six dimensions: decision-making, attitudes towards violence, perceived sexual rights, freedom of mobility, financial independence, and societal norms, and underscores the evolving and multidimensional nature of women’s empowerment in India, revealing significant temporal shifts from 2005−06 to 2019−21. The second-order CFA model was used in this study to capture the evolving nature of women’s empowerment and its dimensions. Traditional factors, such as attitudes towards violence and decision-making, continue to play a vital role, but newer dimensions, such as financial independence and societal norms, are gaining prominence. Further, APC analysis has been used to capture the evolving dimension of women’s empowerment by the women’s age, period and cohort. These changes reflect broader societal transformations and underscore the growing complexity of empowerment in contemporary India.

In 2005−06, freedom of mobility emerged as the most critical dimension of women’s empowerment, highlighting the importance of physical autonomy in a patriarchal society. In the early 2000s, in many parts of India, particularly in rural areas, women’s freedom of mobility was heavily controlled by male family members, reflecting broader societal customs that confined women’s domestic spaces and limited participation in public life [69]. Jejeebhoy and Sathar (2001) identified women’s freedom of mobility as a significant indicator of gender inequality, particularly in South Asia, where women’s roles and behaviour are strictly regulated by cultural norms and customs [21]. They also emphasized that physical mobility is not just about movement, but a precondition for accessing education, employment, and healthcare. Hence, freedom of mobility is not only about physical movement, but it also symbolised broader challenges to the patriarchal norms that sought to control women’s bodies and lives. Several governmental and non-governmental initiatives were designed to promote the education, health, and employment of women in the early 2000s. However, Kishor & Gupta (2004) found that these programs often fail to reach the target population because of the lack of physical accessibility of the services and opportunities [24]. Moreover, Allendorf (2007), noted that women’s ability to visit health facilities or markets without permission was strongly associated with higher autonomy and agency in rural India [38]. Thus, the ability to move freely is an essential factor in gaining autonomy, agency and empowerment.

Decade later, financial independence emerged as the most significant measure of women’s empowerment in India. This change reflects the economic transformation in India. The rapid expansion of financial independence has been driven by increased female labour force participation, evolving socio-economic landscape, and growing emphasis on financial inclusion [47]. Nonetheless, Senarath et al. (2009), demonstrated that women’s access to financial institutions in South Asia began to correlate with increased decision-making power, especially when it allowed for investments in children’s health and education [70]. In India, Desai and Joshi (2019) showed that ownership of mobile phones and bank accounts had measurable effects on women’s intra-household bargaining power [46]. Furthermore, India has witnessed significant policies and interventions for financial inclusion and women’s economic empowerment, such as Pradhan Mantri Jan Dhan Yojna, with the objective of enhancing financial inclusion by providing bank accounts to every household [71]. By the time of 2015−16, women’s participation in the workforce and their contribution to household income became more acceptable, despite the persistence of conventional gender roles in society. This evolving nature of financial independence among women was particularly driven by economic necessity, as families increasingly relied on dual incomes to maintain their standard of living, but it also reflected broader changes in attitudes towards gender equality. Several studies have found that women’s financial contribution to their households can shift dynamics in their favour, leading to greater decision-making authority and empowerment [1,35,63].

In 2019-21, the dimension of attitude towards violence emerged as the most prominent indicator of women’s empowerment. Women’s attitude toward rejecting violence reflects not merely individual agency but a deeper cultural and legal consciousness [17,29]. Nevertheless, the seeds of legal reforms were planted in the early 2000s, including the Protection of Women from Domestic Violence (PWDVA). Despite the existence of the law during the 2005−06 period, its influence was restricted by societal resistance and low awareness. By the time, increased advocacy and education regarding legal rights, women developed a more comprehensive understanding that violence was not only unacceptable but also punishable by law. Studies documented rising rejection of wife-beating in South Asia, particularly in communities exposed to education and media-based interventions [28,48]. Simultaneously, another set of researchers highlights that community-based interventions have created a cultural ripple effect, reshaping what constitutes “acceptable” behaviour in intimate relationships [26,47,69,72]. According to Jejeebhoy et al. (2013), women’s perceptions of empowerment are significantly influenced by their access to justice and legal awareness, which increases their likelihood of rejecting violence. Additionally, numerous studies have shown clear linkages between economic independence and the likelihood of resisting domestic violence [29,30,49]. As women acquired greater autonomy in their lives, their acceptance of violence diminished, and the rejection of violence became a key marker of their empowerment by 2019−21.

Another finding shows a notable trend in the evolving dynamics of women’s age and their empowerment. In 2005−06, older women showed a higher level of empowerment, which can be attributed to their established roles within their families and communities [7,36]. Traditionally, in many parts of India, age and seniority within the family hierarchy confer a certain degree of authority and autonomy to women, particularly after they fulfill expected roles such as childbearing and managing household responsibilities [34]. This autonomy often led towards increased decision-making power and respect within household. However, the trend observed in 2015−16 and 2019−21 presents a remarkable shift in empowerment towards younger women, specifically women aged 25–35 years. These changes can be driven by improved access to education, increased employment opportunities, and evolving social norms regarding marriage and family life. Desai & Andrist (2010), highlighted that educational attainment has increased, particularly for younger-generation women. There has been a corresponding rise in their ability to participate in decision-making processes, both within the household and in the public sphere, which provides the necessary confidence to challenge traditional gender roles and seek greater autonomy. Furthermore, shifting norms around marriage and family life have also contributed to this trend. Srinivasan & James (2015) noted that delayed marriage allows women to complete their education, enter the workforce, and establish themselves economically and socially before taking on the responsibilities of marriage. This delay contributes to higher levels of empowerment among younger women, as they enter marriage with greater resources and a stronger sense of self [41]. Another study revealed that younger women increasingly benefit from the cumulative effects of social reforms and economic opportunities [11,51]. The higher prevalence of employment among young women may also indicate a changing landscape in which traditional markers of the status of empowerment, such as age, are being replaced by factors like education and economic participation [73–75]. Other than the age of the women, our study shows the persistent advantage among women from higher socio-economic groups reflects structural privileges embedded in wealth, place of residence, and social identity [24,56,70,76, 77].

Another key finding from the APC analysis demonstrates that women born in India between 1980 and 1990 exhibit the lowest levels of empowerment when compared to both preceding and succeeding generations, positioning them uniquely as a transitional generation. While this generation benefited from post-liberalization advancements in education and media exposure, these gains did not uniformly translate into enhanced autonomy, mobility, or decision-making authority due to the resilience of restrictive gender norms and familial expectations [21,41]. Unlike earlier cohorts, who gained agency through age and support within extended families. 1980 and 1990 generation came of age amidst the rise of nuclear households, where spousal control replaced traditional in-law authority, often limiting women’s financial and physical freedom [38,76]. The empowerment experienced by this cohort was often instrumental rather than transformative. It was marked by access to education or employment without parallel improvements in sexual autonomy, social independence, or intra-household bargaining power [7,23]. Their limited agency reflects a broader societal disjuncture, while economic liberalization and globalization introduced new opportunities, patriarchal institutions adapted to maintain control over women’s lives, particularly in domains such as marriage, reproductive choices, and public participation [39,46]. In contrast, women born after 1990 entered adulthood in a more progressive environment marked by delayed marriage, better education, opportunity, digital literacy, and greater exposure to rights-based discourse, helping them challenge gender norms more effectively [11].

In light of these findings, it is time to rethink empowerment altogether. Existing frameworks, though insightful, frequently fall short of addressing intricate realities, inherent contradictions, and the dynamic nature of women’s lives in India. Empowerment cannot be reduced to a linear trajectory, nor can it be a one-size-fits-all solution. It is messy, multifaceted, and deeply personal. As we advance our research and policies, acknowledging and embracing this complexity is crucial for truly capturing and addressing the diverse experiences of women in their quest for empowerment.

## Conclusion

This study addresses the critical gap in understanding the evolving nature of women’s empowerment by employing CFA to analyze 27 variables across six dimensions, using three rounds of NFHS data (2005–06, 2015–16, and 2019–21). This approach accounts for the multidimensional nature of women’s empowerment and its temporal changes. Our findings highlight the dynamic nature of empowerment, with shifts in dominant dimensions over time: from “freedom of movement” in 2005–06 to “financial independence” in 2015–16 and “attitude towards violence” by 2019–21. This study also explored another important factor of Indian women’s empowerment and its dimensions with respect to women’s age, period and cohort. Through APC analysis, the study revealed a generational shift, with younger women, particularly those aged 25–35 years, emerging as the most empowered group, in contrast to earlier trends. This underscores the fluidity of empowerment within society. Future research should explore the factors driving the regional and cultural aspects that have led to these shifts.

### Limitations

This study explored six dimensions of empowerment and integrated them into a single construct with strong internal validity. However, it is important to acknowledge the challenges in accurately measuring empowerment. Empowerment is a multifaceted concept that can vary significantly across different regions, cultures, and communities. There are some limitations which must be considered while interpreting the result, such as socially desirable bias could be introduced due to the self-reported data. Another limitation is cultural differences in perception and expression of women empowerment, especially in these two variables “Attitude towards Violence,” and “Perceived Sexual Rights”. These variables are inherently gender-sensitive and often stigmatized within many social contexts, which may lead to underreporting or socially desirable responses and lastly there are no variables available that account for the political dimension of empowerment.

### Recommendations

Based on the above study, the following recommendations can be used to enhance the effectiveness of the existing program and policy

- Implement age-specific strategies: Younger women (15–24) require interventions focused on educational continuity, digital literacy, and reproductive autonomy, while midlife women (25–39) benefit from workplace rights and bargaining power within households. Older women (40+) need enhanced access to healthcare, pensions, and community support networks to sustain autonomy.
- Enhance access to legal aid and grievance redressal: Strengthening legal literacy, expanding women-friendly legal aid cells at the block and panchayat levels, and integrating legal support into women’s helpline services can improve women's attitude towards violence.
- Strengthen rural women’s financial literacy and asset ownership: Programs must integrate financial literacy training, particularly in rural areas, alongside legal support to claim land, property, and inheritance rights.
- Leverage and revitalize community-based platforms: Self-Help Groups (SHGs) should be supported not just as credit groups but as platforms for rights-based training, legal awareness, and collective bargaining. Their roles should be expanded with financial support, capacity building, and institutional linkages.
- Promote regionally contextualized and culturally sensitive programming: Given India’s socio-cultural diversity, empowerment programs should be locally tailored, with community participation, especially in areas with entrenched gender norms.
- Institutionalize strong monitoring and evaluation (M&E) systems: Empowerment programs must include measurable indicators of change in decision-making, mobility, financial control, and bodily autonomy. Model such as Kudumbashree in Kerala provides good M&E practices that can be scaled [78].

## Supporting information

S1 File Covariance structure in CFA. Sample Adequacy test, Exploratory Factor Analysis, Cronbach alpha of each domain for the internal validity of the measure of the women’s empowerment, NFHS-3, NFHS-4 and NFHS-5, India.
(DOCX)

S2 File Construct validity of the indices obtained from confirmatory factor analysis, NFHS-3, NFHS-4 and NFHS-5, India. (DOCX)

S3 File Results of age-period-cohort analysis of women’s empowerment and its dimensions, 2006–2021, India. (DOCX)
