# Supplementary material for: Evolving dimensions of women’s empowerment in India
Source: PLoS One. 2025 Jul 11;20(7):e0327494. doi: 10.1371/journal.pone.0327494 (PMC12250622; doi:10.1371/journal.pone.0327494)
Supplement: S1 File — Sample Adequacy test, Exploratory Factor Analysis, Cronbach alpha of each domain for the internal validity of the measure of the women’s empowerment, NFHS-3, NFHS-4 and NFHS-5, India. (DOCX) [file pone.0327494.s001.docx]

| **Year** | **KMO** |
| --- | --- |
| NFHS 3 | 0.76 |
| NFHS 4 | 0.75 |
| NFHS 5 | 0.73 |

Table1: Kaiser-Meyer-Olkin Measure of Sampling Adequacy, NFHS 3, NFHS 4 and NFHS 5, India

Table 2: variance explained by the retained factor for the women’s empowerment, NFHS 3, India

| **Factor** | **Variance** | **Difference** | **Proportion** | **Cumulative** |
| --- | --- | --- | --- | --- |
|  |  |  |  |  |
| Factor1 | 3.11791 | 0.58521 | 0.1356 | 0.1356 |
| Factor2 | 2.5327 | 0.01562 | 0.1101 | 0.2457 |
| Factor3 | 2.51708 | 0.19793 | 0.1094 | 0.3551 |
| Factor4 | 2.31915 | 0.06342 | 0.1008 | 0.4559 |
| Factor5 | 2.25573 | . | 0.0981 | 0.554 |

Table 3: Factor loading of the retained factors for women’s empowerment index, NFHS 3, India

| **Variable** | **Factor1** | **Factor2** | **Factor3** | **Factor4** | **Factor5** |
| --- | --- | --- | --- | --- | --- |
|  |  |  |  |  |  |
| P1 | -0.0205 | -0.0063 | 0.0057 | -0.0061 | 0.8589 |
| P2 | -0.0101 | 0.0062 | 0.0088 | 0.0096 | 0.888 |
| P3 | 0.0237 | 0.0048 | 0.0037 | 0.0027 | 0.8409 |
| V1 | 0.8048 | -0.0016 | 0.0411 | -0.0237 | -0.0354 |
| V2 | 0.8283 | -0.0471 | 0.01 | -0.0299 | -0.0007 |
| V3 | 0.7991 | 0.009 | 0.0224 | 0.0039 | 0.0044 |
| V4 | 0.7199 | 0.0007 | -0.0242 | 0.0233 | 0.0317 |
| V5 | 0.7694 | 0.0056 | -0.0238 | 0.0255 | -0.0008 |
| D1 | -0.003 | 0.0056 | -0.0322 | 0.7497 | -0.017 |
| D2 | -0.0035 | -0.0286 | 0.0189 | 0.8062 | 0.0117 |
| D3 | -0.0164 | 0.0152 | -0.0024 | 0.7965 | 0.0017 |
| D4 | 0.0149 | -0.0477 | -0.0105 | 0.6971 | 0.0108 |
| M1 | 0.0164 | -0.0087 | 0.8921 | 0.0229 | 0.0296 |
| M2 | 0.024 | -0.0146 | 0.9216 | -0.012 | 0.0046 |
| M3 | -0.012 | 0.0002 | 0.8591 | -0.0321 | -0.0195 |
| F1 | -0.1331 | -0.2244 | 0.2308 | 0.0293 | -0.0055 |
| F2 | 0.0149 | 0.3587 | 0.1841 | 0.0806 | -0.0202 |
| S1 | 0.0841 | 0.7589 | 0.0304 | 0.0074 | 0.001 |
| S2 | 0.0133 | 0.5354 | 0.0542 | 0.0442 | 0.027 |
| S3 | -0.0378 | 0.8681 | -0.0273 | -0.0225 | 0.0014 |
| S4 | -0.0545 | 0.8311 | -0.0351 | -0.0307 | 0.0033 |
| S5 | -0.0364 | 0.4414 | -0.0619 | -0.0445 | 0.0836 |
| S6 | -0.0401 | 0.4284 | 0.0332 | 0.0391 | -0.0893 |

Table 4: Variance explained by the retained factor for the women’s empowerment, NFHS 4, India

| **Factor** | **Variance** | **Difference** | **Proportion** | **Cumulative** |
| --- | --- | --- | --- | --- |
|  |  |  |  |  |
| Factor1 | 3.03486 | 0.18013 | 0.1167 | 0.1167 |
| Factor2 | 2.85473 | 0.26965 | 0.1098 | 0.2265 |
| Factor3 | 2.58508 | 0.13576 | 0.0994 | 0.3259 |
| Factor4 | 2.44932 | 0.01021 | 0.0942 | 0.4202 |
| Factor5 | 2.43911 | 0.67689 | 0.0938 | 0.514 |
| Factor6 | 1.76221 | . | 0.0678 | 0.5817 |

Table5: Factor loading of the retained factors for women’s empowerment index, NFHS 4, India

| **Variable** | **Factor1** | **Factor2** | **Factor3** | **Factor4** | **Factor5** | **Factor6** |
| --- | --- | --- | --- | --- | --- | --- |
|  |  |  |  |  |  |  |
| P1 | -0.0092 | 0.0119 | 0.0191 | 0.0085 | 0.8999 | -0.0108 |
| P2 | -0.0103 | -0.001 | 0.0147 | 0.0168 | 0.9221 | -0.0135 |
| P3 | 0.01 | 0.0063 | 0.01 | 0.0228 | 0.871 | -0.0009 |
| V1 | 0.7937 | -0.0128 | 0.0118 | -0.0194 | -0.0006 | 0.026 |
| V2 | 0.8215 | -0.0057 | 0.0001 | -0.0286 | -0.0144 | 0.0316 |
| V3 | 0.8166 | 0.0039 | 0.0183 | 0.0238 | 0.0144 | 0.0101 |
| V4 | 0.704 | 0.0346 | -0.0007 | 0.0013 | -0.0023 | -0.0372 |
| V5 | 0.7374 | 0.0048 | 0.0164 | 0.0248 | -0.0124 | -0.0425 |
| D1 | 0.0075 | 0.8508 | -0.0306 | -0.014 | 0.0111 | -0.0156 |
| D2 | 0.0036 | 0.8666 | -0.0116 | -0.0138 | -0.0138 | 0.0151 |
| D3 | 0.0069 | 0.8378 | -0.0113 | 0.0043 | -0.0045 | -0.0061 |
| D4 | -0.006 | 0.8166 | -0.0188 | -0.0302 | 0.029 | 0.0288 |
| M1 | 0.0174 | -0.0126 | 0.9034 | -0.0271 | 0.0162 | 0.0005 |
| M2 | 0.0131 | -0.0273 | 0.9219 | -0.0284 | 0.0143 | 0.003 |
| M3 | 0.0102 | -0.0275 | 0.8829 | -0.0607 | 0.0133 | 0.0358 |
| F1 | -0.0035 | 0.023 | 0.0319 | 0.0326 | -0.01 | 0.929 |
| F2 | 0.0133 | -0.0048 | 0.0048 | 0.0316 | -0.0151 | 0.927 |
| F3 | -0.0582 | 0.1104 | 0.2582 | 0.3039 | -0.0457 | 0.3947 |
| F4 | -0.0951 | 0.0792 | 0.1952 | -0.2184 | -0.0058 | 0.4392 |
| F5 | -0.009 | 0.0516 | 0.1464 | 0.0075 | -0.0197 | 0.5421 |
| F6 | -.008 | 0.0002 | 0.2226 | 0.0134 | -0.0087 | 0.5112 |
| S1 | 0.0417 | 0.0148 | 0.0019 | 0.7114 | -0.009 | -0.0721 |
| S2 | -0.0424 | 0.0072 | -0.0044 | 0.4447 | -0.0532 | -0.0562 |
| S3 | 0.0053 | 0.0275 | 0.0965 | 0.3967 | -0.0531 | -0.1279 |
| S4 | -0.015 | -0.039 | -0.0905 | 0.8092 | 0.0271 | 0.0575 |
| S5 | -0.0117 | -0.0444 | -0.0856 | 0.7574 | 0.0474 | 0.0808 |
| S6 | 0.0483 | -0.0376 | -0.0863 | 0.4147 | 0.1252 | 0.0441 |

Table 6 : Variance explained by the retained factor for the women’s empowerment, NFHS 3, India

| **Factor** | **Variance** | **Difference** | **Proportion** | **Cumulative** |
| --- | --- | --- | --- | --- |
|  |  |  |  |  |
| Factor1 | 2.90192 | 0.0686 | 0.1075 | 0.1075 |
| Factor2 | 2.83331 | 0.41268 | 0.1049 | 0.2124 |
| Factor3 | 2.42064 | 0.09135 | 0.0897 | 0.3021 |
| Factor4 | 2.32928 | 0.2043 | 0.0863 | 0.3883 |
| Factor5 | 2.12498 | 0.35857 | 0.0787 | 0.467 |
| Factor6 | 1.76641 | . | 0.0654 | 0.5325 |

Table 7: Factor loading of the retained factors for women’s empowerment index, NFHS 5, India

| **Variable** | **Factor1** | **Factor2** | **Factor3** | **Factor4** | **Factor5** | **Factor6** |
| --- | --- | --- | --- | --- | --- | --- |
|  |  |  |  |  |  |  |
| P1 | -0.0227 | -0.0409 | -0.0487 | -0.0011 | 0.8379 | -0.0329 |
| P2 | 0.0055 | -0.0314 | -0.0748 | -0.022 | 0.8686 | -0.0016 |
| P3 | 0.018 | -0.0024 | -0.0253 | -0.0294 | 0.7782 | 0.0545 |
| V1 | 0.7655 | 0.0097 | -0.0157 | 0.0023 | 0.0073 | -0.013 |
| V2 | 0.7804 | -0.0005 | -0.0233 | 0.0108 | -0.0558 | 0.0295 |
| V3 | 0.7991 | 0.0095 | 0.035 | 0.0002 | -0.0162 | 0.0075 |
| V4 | 0.7164 | -0.0014 | 0.0123 | 0.0005 | 0.0558 | -0.0099 |
| V5 | 0.7258 | -0.0026 | 0.0147 | -0.0071 | 0.0249 | -0.0302 |
| D1 | 0.0119 | 0.841 | -0.0324 | -0.0103 | -0.0164 | -0.0248 |
| D2 | -0.0051 | 0.8613 | -0.0209 | -0.0207 | -0.0303 | 0.0105 |
| D3 | 0.0088 | 0.8355 | -0.0087 | -0.0051 | -0.0198 | -0.0156 |
| D4 | -0.0008 | 0.7993 | -0.0288 | -0.0171 | -0.0132 | 0.0179 |
| M1 | 0.0186 | 0.0031 | -0.0215 | 0.853 | -0.0274 | 0.0201 |
| M2 | -0.0116 | -0.0324 | -0.0052 | 0.9083 | -0.0088 | -0.0117 |
| M3 | 0.0028 | -0.0174 | -0.0251 | 0.8661 | -0.0133 | -0.0186 |
| F1 | -0.0134 | 0.0099 | 0.0317 | -0.005 | 0.0145 | 0.9246 |
| F2 | 0.0072 | -0.0217 | 0.0122 | -0.0064 | -0.0055 | 0.9236 |
| F3 | -0.0388 | 0.1151 | 0.1702 | 0.085 | 0.153 | 0.4747 |
| F4 | -0.1127 | 0.0877 | -0.0683 | 0.0618 | 0.0469 | 0.4114 |
| F5 | -0.0132 | 0.0742 | 0.1857 | 0.0291 | 0.0712 | 0.5224 |
| F6 | -0.0023 | 0.0345 | 0.2450 | 0.0012 | 0.0567 | 0.5386 |
| S1 | 0.0263 | -0.0173 | 0.6912 | -0.0423 | 0.0603 | -0.0882 |
| S2 | -0.0657 | 0.0046 | 0.457 | -0.0184 | -0.0218 | -0.07 |
| S3 | -0.0238 | 0.0149 | 0.4127 | 0.0379 | 0.1034 | -0.1104 |
| S4 | 0.0048 | -0.0424 | 0.8442 | -0.0156 | -0.0906 | 0.0402 |
| S5 | 0.007 | -0.0358 | 0.8065 | -0.0098 | -0.0984 | 0.0691 |
| S6 | 0.0568 | -0.0185 | 0.4478 | 0.0047 | -0.0256 | 0.1092 |

Table 8: Cronbach alpha of each domain for the internal validity of the measure of the women’s empowerment, NFHS 3, NFHS 4 and NFHS 5, India

| **Dimensions** | **NFHS 3** | **NFHS 4** | **NFHS 5** |
| --- | --- | --- | --- |
| Mobility | 0.87 | 0.89 | 0.86 |
| ATV | 0.83 | 0.84 | 0.82 |
| Decision making | 0.75 | 0.86 | 0.86 |
| PSR | 0.83 | 0.87 | 0.78 |
| Societal Norms | 0.67 | 0.61 | 0.62 |
| Financial freedom | nil | 0.65 | 0.68 |
