# Supplementary material for: Evolving dimensions of women’s empowerment in India
Source: PLoS One. 2025 Jul 11;20(7):e0327494. doi: 10.1371/journal.pone.0327494 (PMC12250622; doi:10.1371/journal.pone.0327494)
Supplement: S2 File — (DOCX) [file pone.0327494.s002.docx]

**S2 Table:** Construct validity of the indices obtained from confirmatory factor analysis, 2005-06, 2015-16 and 2019-2021, India

| Survey | RMSEA | CFI | TLI | SRMR |
| --- | --- | --- | --- | --- |
|  |  |  |  |  |
| 2005-06 | 0.018 | 0.994 | 0.992 | 0.015 |
| 2015-16 | 0.010 | 0.989 | 0.995 | 0.010 |
| 2019-21 | 0.008 | 0.995 | 0.994 | 0.007 |
| RMSEA: Root mean squared error of approximation | | | | |
| CFI: Comparative fit index | | |  |  |
| TLI: Tucker–Lewis index | | |  |  |
| SRMR: Standardized root mean squared residual | | | | |
