# Supplementary material for: Evolving dimensions of women’s empowerment in India
Source: PLoS One. 2025 Jul 11;20(7):e0327494. doi: 10.1371/journal.pone.0327494 (PMC12250622; doi:10.1371/journal.pone.0327494)
Supplement: S3 File — (DOCX) [file pone.0327494.s003.docx]

**S3 Table:** Results of age-period-cohort analysis of women’s empowerment and its dimensions, 2006 to 2021, India

|  | **Empowerment** | **Decision-Making** | **PSR** | **Freedom of Mobility** | **Societal Norms** | **ATV** |
| --- | --- | --- | --- | --- | --- | --- |
| Age-Period-Cohort | Coef (95% CI) | Coef (95% CI) | Coef (95% CI) | Coef (95% CI) | Coef (95% CI) | coef (95% CI) |
|  |  |  |  |  |  |  |
| Age16 | 0.13*(0.02,0.23) | 0.06(-0.08,0.20) | 0.01(-0.10,0.11) | 0.07(-0.05,0.20) | 0.08(-0.07,0.23) | 0.08(-0.07,0.23) |
| Age17 | 0.28***(0.18,0.38) | 0.18**(0.05,0.31) | 0.07(-0.04,0.17) | 0.15*(0.03,0.27) | 2.26***(1.49,3.03) | 0.10(-0.03,0.24) |
| Age18 | 0.39***(0.30,0.48) | 0.24***(0.12,0.36) | 0.09(-0.01,0.18) | 0.19**(0.08,0.30) | 3.27***(2.55,3.99) | 0.11(-0.02,0.24) |
| Age19 | 0.49***(0.4,0.58) | 0.33***(0.21,0.44) | 0.08(-0.02,0.17) | 0.30***(0.19,0.41) | 4.11***(3.4,4.82) | 0.14*(0.01,0.26) |
| Age20 | 0.56***(0.47,0.65) | 0.40***(0.28,0.52) | 0.08(-0.02,0.17) | 0.41***(0.31,0.52) | 4.20***(3.5,4.9) | 0.10(-0.02,0.23) |
| Age21 | 0.69***(0.59,0.78) | 0.53***(0.41,0.65) | 0.10*(0.01,0.19) | 0.49***(0.39,0.60) | 5.01***(4.3,5.71) | 0.14*(0.01,0.27) |
| Age22 | 0.73***(0.63,0.82) | 0.56***(0.44,0.68) | 0.13**(0.03,0.23) | 0.60***(0.49,0.71) | 5.12***(4.41,5.83) | 0.14*(0.02,0.27) |
| Age23 | 0.79***(0.70,0.89) | 0.64***(0.51,0.76) | 0.12*(0.03,0.22) | 0.70***(0.59,0.81) | 5.63***(4.92,6.34) | 0.15*(0.03,0.28) |
| Age24 | 0.84***(0.74,0.93) | 0.70***(0.58,0.83) | 0.11*(0.01,0.21) | 0.77***(0.66,0.88) | 5.71***(4.99,6.43) | 0.16*(0.03,0.29) |
| Age25 | 0.81***(0.71,0.9) | 0.73***(0.61,0.85) | 0.09(0.01,0.19) | 0.80***(0.69,0.91) | 5.22***(4.49,5.94) | 0.13(0.01,0.26) |
| Age26 | 0.89***(0.79,0.98) | 0.77***(0.64,0.9) | 0.12*(0.02,0.22) | 0.90***(0.78,1.01) | 5.88***(5.14,6.63) | 0.14*(0.01,0.27) |
| Age27 | 0.90***(0.80,1.00) | 0.80***(0.67,0.93) | 0.12*(0.02,0.22) | 0.94***(0.82,1.05) | 5.78***(5.01,6.54) | 0.14*(0.01,0.28) |
| Age28 | 0.89***(0.79,0.99) | 0.84***(0.71,0.97) | 0.11*(0.01,0.22) | 0.94***(0.82,1.06) | 5.46***(4.69,6.23) | 0.12(-0.01,0.26) |
| Age29 | 0.94***(0.84,1.05) | 0.87***(0.73,1) | 0.11*(0.01,0.22) | 1.00***(0.87,1.12) | 5.95***(5.16,6.75) | 0.15*(0.01,0.29) |
| Age30 | 0.86***(0.76,0.97) | 0.85***(0.71,0.99) | 0.10(-0.01,0.21) | 0.96***(0.84,1.09) | 4.63***(3.82,5.43) | 0.09(-0.06,0.23) |
| Age31 | 0.95***(0.84,1.06) | 0.91***(0.77,1.06) | 0.12*(0.01,0.24) | 1.00***(0.87,1.13) | 5.76***(4.92,6.59) | 0.11(-0.04,0.26) |
| Age32 | 0.91***(0.79,1.02) | 0.89***(0.74,1.04) | 0.11(-0.01,0.23) | 0.99***(0.86,1.12) | 4.96***(4.11,5.82) | 0.08(-0.08,0.23) |
| Age33 | 0.92***(0.81,1.04) | 0.92***(0.77,1.07) | 0.1(-0.02,0.23) | 0.99***(0.86,1.13) | 5.3***(4.42,6.18) | 0.08(-0.08,0.24) |
| Age34 | 0.92***(0.80,1.04) | 0.92***(0.77,1.08) | 0.12(-0.01,0.24) | 0.98***(0.83,1.12) | 4.97***(4.06,5.87) | 0.08(-0.08,0.24) |
| Age35 | 0.84***(0.72,0.96) | 0.9***(0.74,1.06) | 0.09(-0.03,0.22) | 0.95***(0.81,1.10) | 3.86***(2.93,4.78) | 0.01(-0.16,0.17) |
| Age36 | 0.89***(0.76,1.01) | 0.92***(0.75,1.08) | 0.11(-0.02,0.24) | 0.94***(0.79,1.09) | 4.40***(3.45,5.36) | 0.07(-0.10,0.24) |
| Age37 | 0.87***(0.74,1.00) | 0.94***(0.77,1.11) | 0.11(-0.02,0.25) | 0.93***(0.78,1.09) | 4.21***(3.23,5.2) | 0.05(-0.13,0.22) |
| Age38 | 0.83***(0.70,0.96) | 0.93***(0.75,1.1) | 0.10(-0.04,0.24) | 0.90***(0.74,1.06) | 3.74***(2.73,4.75) | 0.02(-0.16,0.20) |
| Age39 | 0.84***(0.70,0.97) | 0.92***(0.74,1.1) | 0.12(-0.03,0.26) | 0.91***(0.74,1.07) | 3.78***(2.74,4.82) | 0.01(-0.18,0.19) |
| Age40 | 0.70***(0.56,0.84) | 0.87***(0.69,1.06) | 0.08(-0.07,0.23) | 0.85***(0.69,1.02) | 2.37***(1.31,3.44) | 0.01(-0.18,0.20) |
| Age41 | 0.75***(0.61,0.89) | 0.93***(0.74,1.12) | 0.08(-0.07,0.24) | 0.88***(0.71,1.06) | 3.21***(2.1,4.32) | -0.01(-0.20,0.19) |
| Age42 | 0.71***(0.56,0.85) | 0.91***(0.71,1.10) | 0.12(-0.03,0.28) | 0.86***(0.68,1.04) | 2.51***(1.38,3.64) | -0.05(-0.25,0.15) |
| Age43 | 0.7.0***(0.54,0.85) | 0.88***(0.67,1.08) | 0.12(-0.04,0.28) | 0.83***(0.64,1.01) | 2.70***(1.54,3.87) | -0.04(-0.25,0.17) |
| Age44 | 0.68***(0.53,0.84) | 0.86***(0.65,1.07) | 0.12(-0.04,0.29) | 0.79***(0.60,0.97) | 2.70***(1.51,3.9) | -0.06(-0.27,0.15) |
| Age45 | 0.57***(0.41,0.73) | 0.81***(0.6,1.02) | 0.07(-0.1,0.24) | 0.76***(0.57,0.95) | 1.66**(0.44,2.88) | -0.09(-0.31,0.13) |
| Age46 | 0.62***(0.46,0.79) | 0.84***(0.62,1.06) | 0.10(-0.08,0.27) | 0.69***(0.5,0.89) | 2.17**(0.91,3.43) | -0.05(-0.28,0.17) |
| Age47 | 0.61***(0.44,0.77) | 0.87***(0.65,1.10) | 0.09(-0.1,0.27) | 0.71***(0.51,0.91) | 2.04**(0.75,3.34) | -0.08(-0.31,0.15) |
| Age48 | 0.58***(0.41,0.75) | 0.79***(0.56,1.02) | 0.11(-0.08,0.29) | 0.66***(0.46,0.87) | 1.97**(0.65,3.29) | -0.09(-0.33,0.14) |
| Age49 | 0.55***(0.38,0.73) | 0.76***(0.53,0.99) | 0.07(-0.12,0.26) | 0.65***(0.44,0.86) | 1.89**(0.56,3.22) | -0.09(-0.33,0.14) |
| Year2006 | 0.26***(0.24,0.28) | 0.14***(0.11,0.17) | 0.27***(0.25,0.3) | 0.25***(0.22,0.27) | 1.57***(1.4,1.73) | 0.11***(0.09,0.14) |
| Year2015 | 0.08**(0.03,0.13) | 0.67***(0.60,0.74) | 0.19***(0.13,0.24) | 0.38***(0.31,0.44) | -3.86***(-4.27,-3.46) | 0.22***(0.15,0.29) |
| Year2016 | 0.33***(0.27,0.38) | 0.75***(0.68,0.83) | 0.39***(0.33,0.45) | 0.35***(0.28,0.42) | -3.44***(-3.88,-2.99) | 0.52***(0.44,0.60) |
| Year2019 | 0.14***(0.07,0.21) | 0.84***(0.74,0.93) | 0.02(-0.05,0.10) | 1.29***(1.21,1.38) | -6.88***(-7.43,-6.33) | 0.42***(0.33,0.52) |
| Year2020 | 0.18***(0.1,0.26) | 0.81***(0.70,0.91) | 0.05(-0.03,0.13) | 1.28***(1.19,1.38) | -6.9***(-7.49,-6.31) | 0.51***(0.40,0.61) |
| Year2021 | 0.27***(0.19,0.35) | 0.96***(0.85,1.06) | 0.03(-0.06,0.12) | 1.31***(1.22,1.41) | -6.67***(-7.29,-6.04) | 0.62***(0.51,0.73) |
| Cohort1957 | 0.14(-0.08,0.35) | -0.08(-0.36,0.21) | 0.02(-0.21,0.26) | 0.24(-0.02,0.50) | 2.27**(0.61,3.93) | 0.39**(0.10,0.69) |
| Cohort1958 | 0.09(-0.12,0.3) | -0.12(-0.40,0.16) | -0.02(-0.25,0.21) | 0.20(-0.06,0.45) | 1.72*(0.09,3.35) | 0.35*(0.06,0.64) |
| Cohort1959 | 0.04(-0.16,0.25) | -0.19(-0.46,0.09) | -0.01(-0.24,0.21) | 0.16(-0.09,0.41) | 1.56(-0.04,3.16) | 0.32*(0.03,0.61) |
| Cohort1960 | 0.05(-0.15,0.25) | -0.14(-0.42,0.13) | -0.03(-0.25,0.19) | 0.18(-0.07,0.42) | 1.57*(0.02,3.13) | 0.28(0.01,0.56) |
| Cohort1961 | 0.05(-0.15,0.24) | -0.14(-0.4,0.12) | 0.01(-0.2,0.23) | 0.07(-0.16,0.31) | 1.22(-0.29,2.73) | 0.28*(0.01,0.55) |
| Cohort1962 | 0.01(-0.19,0.20) | -0.17(-0.43,0.09) | -0.04(-0.25,0.17) | 0.08(-0.15,0.31) | 1.08(-0.41,2.56) | 0.33*(0.07,0.6) |
| Cohort1963 | -0.03(-0.21,0.16) | -0.17(-0.42,0.08) | -0.02(-0.22,0.18) | 0.01(-0.23,0.23) | 1.48*(0.04,2.93) | 0.34*(0.08,0.59) |
| Cohort1964 | -0.06(-0.24,0.13) | -0.23(-0.48,0.01) | -0.04(-0.24,0.16) | -0.02(-0.24,0.20) | 1.32(-0.09,2.73) | 0.29*(0.04,0.54) |
| Cohort1965 | -0.06(-0.24,0.12) | -0.22(-0.46,0.02) | -0.01(-0.20,0.19) | -0.04(-0.25,0.18) | 0.97(-0.4,2.34) | 0.31*(0.06,0.55) |
| Cohort1966 | -0.16(-0.33,0.01) | -0.20(-0.43,0.03) | 0.01(-0.18,0.19) | -0.07(-0.27,0.14) | 0.49(-0.82,1.8) | 0.16(-0.08,0.39) |
| Cohort1967 | -0.25**(-0.41,-0.08) | -0.23*(-0.46,-0.01) | -0.03(-0.21,0.15) | -0.06(-0.26,0.14) | -0.2(-1.47,1.08) | 0.26*(0.03,0.49) |
| Cohort1968 | -0.25**(-0.41,-0.09) | -0.24*(-0.46,-0.02) | 0.01(-0.18,0.17) | -0.08(-0.28,0.11) | -0.15(-1.39,1.09) | 0.20(-0.03,0.42) |
| Cohort1969 | -0.24**(-0.4,-0.08) | -0.28*(-0.49,-0.07) | 0.01(-0.17,0.17) | -0.11(-0.30,0.08) | -0.05(-1.26,1.16) | 0.22*(0.01,0.44) |
| Cohort1970 | -0.27**(-0.42,-0.12) | -0.25*(-0.46,-0.05) | -0.01(-0.17,0.16) | -0.16(-0.34,0.02) | -0.23(-1.39,0.93) | 0.22*(0.01,0.42) |
| Cohort1971 | -0.29***(-0.44,-0.15) | -0.26**(-0.46,-0.07) | 0.01(-0.15,0.17) | -0.22*(-0.40,-0.05) | -0.42(-1.55,0.71) | 0.17(-0.03,0.37) |
| Cohort1972 | -0.29***(-0.43,-0.14) | -0.29**(-0.48,-0.10) | -0.01(-0.16,0.15) | -0.25**(-0.43,-0.08) | -0.20(-1.29,0.90) | 0.20*(0.01,0.39) |
| Cohort1973 | -0.29***(-0.42,-0.15) | -0.29**(-0.48,-0.11) | 0.01(-0.15,0.14) | -0.27**(-0.44,-0.10) | -0.14(-1.2,0.92) | 0.21*(0.02,0.40) |
| Cohort1974 | -0.28***(-0.42,-0.15) | -0.29**(-0.47,-0.11) | 0.01(-0.15,0.14) | -0.29***(-0.45,-0.13) | -0.21(-1.24,0.82) | 0.21*(0.03,0.39) |
| Cohort1975 | -0.34***(-0.47,-0.21) | -0.33***(-0.50,-0.16) | 0.01(-0.14,0.14) | -0.32***(-0.48,-0.17) | -0.66(-1.65,0.33) | 0.16(-0.02,0.33) |
| Cohort1976 | -0.35***(-0.47,-0.22) | -0.31***(-0.48,-0.15) | 0.01(-0.13,0.14) | -0.36***(-0.51,-0.21) | -0.58(-1.53,0.37) | 0.15(-0.02,0.32) |
| Cohort1977 | -0.37***(-0.49,-0.25) | -0.35***(-0.51,-0.19) | -0.01(-0.14,0.12) | -0.39***(-0.54,-0.25) | -0.64(-1.56,0.28) | 0.18*(0.01,0.34) |
| Cohort1978 | -0.39***(-0.51,-0.28) | -0.37***(-0.52,-0.21) | -0.01(-0.14,0.11) | -0.42***(-0.56,-0.28) | -0.90*(-1.79,-0.01) | 0.16(0,0.32) |
| Cohort1979 | -0.38***(-0.49,-0.27) | -0.34***(-0.49,-0.19) | 0.01(-0.12,0.12) | -0.43***(-0.56,-0.29) | -0.93*(-1.79,-0.08) | 0.15*(0,0.31) |
| Cohort1980 | -0.41***(-0.52,-0.31) | -0.37***(-0.51,-0.23) | 0.01(-0.10,0.13) | -0.46***(-0.59,-0.33) | -1.21**(-2.03,-0.39) | 0.14(0.01,0.29) |
| Cohort1981 | -0.45***(-0.55,-0.35) | -0.39***(-0.52,-0.25) | -0.02(-0.14,0.09) | -0.49***(-0.62,-0.37) | -1.22**(-2.01,-0.43) | 0.12(-0.02,0.26) |
| Cohort1982 | -0.45***(-0.55,-0.35) | -0.40***(-0.54,-0.27) | 0.01(-0.11,0.10) | -0.51***(-0.63,-0.39) | -1.23**(-1.99,-0.47) | 0.12(-0.01,0.26) |
| Cohort1983 | -0.46***(-0.56,-0.37) | -0.40***(-0.53,-0.28) | 0.01(-0.10,0.10) | -0.53***(-0.64,-0.41) | -1.64***(-2.37,-0.92) | 0.12(-0.01,0.25) |
| Cohort1984 | -0.47***(-0.56,-0.38) | -0.40***(-0.52,-0.28) | -0.01(-0.1,0.09) | -0.55***(-0.66,-0.44) | -1.57***(-2.27,-0.88) | 0.12(0,0.25) |
| Cohort1985 | -0.48***(-0.56,-0.39) | -0.43***(-0.55,-0.31) | 0.01(-0.08,0.10) | -0.56***(-0.67,-0.46) | -1.77***(-2.44,-1.10) | 0.11(-0.01,0.23) |
| Cohort1986 | -0.49***(-0.57,-0.41) | -0.41***(-0.52,-0.30) | 0.01(-0.09,0.08) | -0.59***(-0.69,-0.49) | -1.82***(-2.46,-1.18) | 0.08(-0.03,0.19) |
| Cohort1987 | -0.46***(-0.54,-0.38) | -0.42***(-0.53,-0.31) | 0.01(-0.09,0.08) | -0.62***(-0.72,-0.53) | -1.66***(-2.28,-1.05) | 0.09(-0.02,0.20) |
| Cohort1988 | -0.48***(-0.55,-0.40) | -0.44***(-0.54,-0.34) | 0.01(-0.07,0.10) | -0.62***(-0.71,-0.53) | -1.98***(-2.57,-1.39) | 0.07(-0.03,0.18) |
| Cohort1989 | -0.45***(-0.52,-0.37) | -0.42***(-0.52,-0.32) | 0.01(-0.08,0.08) | -0.65***(-0.74,-0.57) | -1.77***(-2.33,-1.20) | 0.08(-0.02,0.18) |
| Cohort1990 | -0.44***(-0.51,-0.37) | -0.43***(-0.53,-0.34) | 0.02(-0.06,0.09) | -0.67***(-0.75,-0.58) | -1.85***(-2.39,-1.31) | 0.06(-0.04,0.15) |
| Cohort1991 | -0.41***(-0.48,-0.35) | -0.46***(-0.54,-0.37) | 0.02(-0.05,0.09) | -0.67***(-0.75,-0.59) | -1.66***(-2.17,-1.15) | 0.07(-0.02,0.16) |
| Cohort1992 | -0.41***(-0.48,-0.34) | -0.41***(-0.49,-0.32) | 0.02(-0.05,0.08) | -0.68***(-0.76,-0.60) | -1.93***(-2.44,-1.42) | 0.04(-0.05,0.14) |
| Cohort1993 | -0.39***(-0.45,-0.33) | -0.39***(-0.47,-0.3) | 0.01(-0.05,0.08) | -0.65***(-0.73,-0.57) | -1.85***(-2.34,-1.36) | 0.04(-0.04,0.13) |
| Cohort1994 | -0.38***(-0.45,-0.32) | -0.36***(-0.44,-0.28) | 0.01(-0.07,0.06) | -0.60***(-0.68,-0.53) | -2.09***(-2.57,-1.6) | 0.07(-0.02,0.16) |
| Cohort1995 | -0.35***(-0.42,-0.29) | -0.35***(-0.43,-0.26) | 0.02(-0.04,0.09) | -0.59***(-0.66,-0.51) | -1.94***(-2.41,-1.47) | 0.06(-0.02,0.15) |
| Cohort1996 | -0.33***(-0.39,-0.27) | -0.34***(-0.42,-0.26) | 0.04(-0.03,0.10) | -0.51***(-0.58,-0.44) | -2.03***(-2.5,-1.56) | 0.04(-0.04,0.13) |
| Cohort1997 | -0.33***(-0.4,-0.27) | -0.35***(-0.43,-0.27) | -0.01(-0.07,0.06) | -0.47***(-0.55,-0.4) | -2.17***(-2.64,-1.69) | 0.04(-0.04,0.13) |
| Cohort1998 | -0.32***(-0.39,-0.26) | -0.33***(-0.42,-0.25) | 0.01(-0.06,0.07) | -0.40***(-0.48,-0.33) | -2.15***(-2.63,-1.66) | 0.04(-0.05,0.13) |
| Cohort1999 | -0.29***(-0.35,-0.22) | -0.30***(-0.39,-0.21) | 0.03(-0.04,0.09) | -0.27***(-0.35,-0.19) | -2.23***(-2.73,-1.72) | 0.04(-0.05,0.13) |
| Cohort2000 | -0.28***(-0.35,-0.21) | -0.30***(-0.39,-0.21) | -0.01(-0.08,0.06) | -0.20***(-0.28,-0.12) | -2.29***(-2.82,-1.76) | 0.08(-0.01,0.17) |
| Cohort2001 | -0.22***(-0.29,-0.14) | -0.21***(-0.31,-0.12) | 0.02(-0.05,0.10) | -0.13**(-0.21,-0.04) | -2.20***(-2.77,-1.64) | 0.03(-0.07,0.13) |
| Cohort2002 | -0.17***(-0.26,-0.08) | -0.23***(-0.35,-0.11) | 0.04(-0.06,0.13) | -0.08(-0.19,0.03) | -2.26***(-2.96,-1.55) | 0.05(-0.08,0.17) |
| Cohort2003 | -0.16**(-0.27,-0.05) | -0.21**(-0.36,-0.06) | 0.01(-0.11,0.12) | 0.09(-0.05,0.22) | -2.24***(-3.12,-1.35) | -0.08(-0.24,0.07) |
| Cohort2004 | -0.14(-0.31,0.04) | -0.29*(-0.52,-0.05) | -0.1(-0.28,0.08) | 0.03(-0.18,0.24) | -1.04(-2.37,0.3) | -0.06(-0.30,0.17) |
| Cohort2005 | -0.15(-0.51,0.22) | -0.24(-0.74,0.25) | 0.02(-0.34,0.38) | 0.25(-0.19,0.69) | -0.37(-3.2,2.46) | -0.03(-0.54,0.48) |
| Cohort2006 | 0.12(-0.50,0.73) | -0.19(-0.99,0.61) | 0.23(-0.37,0.83) | 0.49(-0.25,1.22) | -1.07(-5.77,3.63) | 0.05(-0.77,0.87) |
| _cons | 1.45***(1.37,1.52) | 1.15***(1.05,1.26) | 1.66***(1.58,1.75) | 0.63***(0.54,0.73) | 19.84***(19.23,20.45) | 2.11***(2,2.22) |

* p<0.05, ** p<0.01, *** p<0.001

PSR: Perceived Sexual Rights

ATV: Attitude towards violence

Coef: coefficient

CI: confidence interval
